# High Molecular Weight Fibroblast Growth Factor-2 in the Human Heart Is a Potential Target for Prevention of Cardiac Remodeling

**DOI:** 10.1371/journal.pone.0097281

**Published:** 2014-05-14

**Authors:** Jon-Jon Santiago, Leslie J. McNaughton, Navid Koleini, Xin Ma, Brian Bestvater, Barbara E. Nickel, Robert R. Fandrich, Jeffrey T. Wigle, Darren H. Freed, Rakesh C. Arora, Elissavet Kardami

**Affiliations:** 1 Institute of Cardiovascular Sciences, St. Boniface Hospital Research Centre, University of Manitoba, Winnipeg, Manitoba, Canada; 2 Department of Human Anatomy & Cell Sciences, University of Manitoba, Winnipeg, Manitoba, Canada; 3 Department of Physiology, University of Manitoba, Winnipeg, Manitoba, Canada; 4 Department of Biochemistry and Medical Genetics, University of Manitoba, Winnipeg, Manitoba, Canada; 5 Department of Surgery, University of Manitoba, Winnipeg, Manitoba, Canada; San Diego State University, United States of America

## Abstract

Fibroblast growth factor 2 (FGF-2) is a multifunctional protein synthesized as high (Hi-) and low (Lo-) molecular weight isoforms. Studies using rodent models showed that Hi- and Lo-FGF-2 exert distinct biological activities: after myocardial infarction, rat Lo-FGF-2, but not Hi-FGF-2, promoted sustained cardioprotection and angiogenesis, while Hi-FGF-2, but not Lo-FGF-2, promoted myocardial hypertrophy and reduced contractile function. Because there is no information regarding Hi-FGF-2 in human myocardium, we undertook to investigate expression, regulation, secretion and potential tissue remodeling-associated activities of human cardiac (atrial) Hi-FGF-2. Human patient-derived atrial tissue extracts, as well as pericardial fluid, contained Hi-FGF-2 isoforms, comprising, respectively, 53%(±20 SD) and 68% (±25 SD) of total FGF-2, assessed by western blotting. Human atrial tissue-derived primary myofibroblasts (hMFs) expressed and secreted predominantly Hi-FGF-2, at about 80% of total. Angiotensin II (Ang II) up-regulated Hi-FGF-2 in hMFs, via activation of both type 1 and type 2 Ang II receptors; the ERK pathway; and matrix metalloprotease-2. Treatment of hMFs with neutralizing antibodies selective for human Hi-FGF-2 (neu-Ab^Hi-FGF-2^) reduced accumulation of proteins associated with fibroblast-to-myofibroblast conversion and fibrosis, including α-smooth muscle actin, extra-domain A fibronectin, and procollagen. Stimulation of hMFs with recombinant human Hi-FGF-2 was significantly more potent than Lo-FGF-2 in upregulating inflammation-associated proteins such as pro-interleukin-1β and plasminogen-activator-inhibitor-1. Culture media conditioned by hMFs promoted cardiomyocyte hypertrophy, an effect that was prevented by neu-Ab^Hi-FGF-2^ *in vitro*. In conclusion, we have documented that Hi-FGF-2 represents a substantial fraction of FGF-2 in human cardiac (atrial) tissue and in pericardial fluid, and have shown that human Hi-FGF-2, unlike Lo-FGF-2, promotes deleterious (pro-fibrotic, pro-inflammatory, and pro-hypertrophic) responses *in vitro.* Selective targeting of Hi-FGF-2 production may, therefore, reduce pathological remodelling in the human heart.

## Introduction

Chronic ischemic heart disease, hypertension, and various types of cardiomyopathies are characterized by maladaptive changes leading to heart failure. These changes can include cardiomyocyte hypertrophy, enhanced innate inflammation, and transformation of fibroblasts and potentially other cell types to a myofibroblast phenotype promoting fibrosis [1], [2]. Understanding the cellular and molecular mechanisms contributing to cardiac remodelling can lead to new approaches for prevention, reversal, or management of pathological changes and thus improve cardiac outcome. Cytokines and growth factors, secreted into the extracellular and interstitial space by cardiac cells, promote, as well as sustain, cardiac inflammasome activation, fibrosis, and hypertrophy[2], [3]. One such growth factor, expressed by both myocytes and non-myocytes, is fibroblast growth factor-2 (FGF-2) [4]. FGF-2-null mouse models demonstrated that FGF-2, secreted by cardiac non-myocytes, mediated the development of cardiac hypertrophy in response to pressure overload or elevated Angiotensin II levels [5], [6]. Fibroblast-produced FGF-2 was strongly implicated in the induction of fibrosis in a mouse model of pressure overload [7].

Fibroblast growth factor 2 (FGF-2) is a member of the larger family of heparin-binding growth factors, and is synthesized by cells as as high molecular weight (>20 kDa, Hi-) or low molecular weight (18 kDa, Lo-) isoforms from a single mRNA, translated, respectively, from CUG or AUG start sites [8]. A variety of stress stimuli including oxidative stress and heat shock have been reported to favor translation from CUG sites and accumulation of Hi-FGF-2 isoforms[9]. FGF-2 is found in the intracellular as well as extracellular environment and is capable of activating intracellular (intracrine) as well as paracrine and autocrine signaling pathways[8]. Although FGF-2 lacks a classic signal peptide sequence it is nevertheless released to the extracellular space by non-conventional secretory pathways, and as a consequence of cellular injury, transient or irreversible[10], [11]. Hi-FGF-2 is often referred to as ‘the nuclear’ FGF-2, and has traditionally been considered to exert exclusively nuclear activities[12]. The paracrine or autocrine activities of exported FGF-2 have been attributed to Lo-FGF-2, considered to be the only FGF-2 isoform secreted to the extracellular environment [13] [12]. There is, however, increasing evidence that Hi-FGF-2 is also exported/secreted from cells and can exert distinct effects compared to those induced by Lo-FGF-2. For example, Lo-FGF-2 promotes, while Hi-FGF-2 inhibits, endothelial cell migration and angiogenesis[14], [15], [16]. In addition, administration of rat Hi-FGF-2, but not Lo-FGF-2 after myocardial infarction promoted cardiomyocyte and cardiac hypertrophy; in the same model, rat Lo-FGF-2, but not Hi-FGF-2, was capable of sustained cardioprotection and angiogenesis after myocardiac infarction[17]. The exaggerated cardiac hypertrophy and fibrosis observed in a mouse model subjected to pressure overload correlated with significantly elevated cardiac Hi-FGF-2 levels, pointing to Hi-, rather than Lo-FGF-2 as an agent of pathological change[18]. Rat cardiac myofibroblasts were documented to predominantly express and secrete Hi-FGF-2, by a caspase-1-dependent mechanism, implicating Hi-FGF-2 in the innate inflammation response [19]. Finally, transgenic mouse models engineered to express only Hi- or only Lo-FGF-2 have shown that Hi-FGF-2 expression was associated with increased susceptibility to ischemia and reperfusion injury while Lo-FGF-2 exerted a protective effect [20], [21]. Taken together, information from animal models supports the notion that unlike Lo-FGF-2, Hi-FGF-2 exerts deleterious effects in the heart, and makes a compelling case for determining the potential role as well as relevance of Hi-FGF-2 in the human heart.

In this work, we examined relative expression of FGF-2 isoforms in human atrial tissue and pericardial fluid as well as in atrial tissue-derived myofibroblast primary cultures (hMFs). In addition, as angiotensin II (Ang II) is a potent bioactive agent which mediates pathological cellular changes in several types of chronic heart disease [22], [23], [24], we investigated the regulation of human cardiac Hi-FGF-2 production by Ang II (and associated signal transduction pathways) in hMFs. Stimulation of hMFs with recombinant human FGF-2 isoforms was used to examine effects on innate inflammation-associated proteins such as pro-interleukin 1β (pro-IL-1β) and tissue plasminogen activator inhibitor 1 (PAI-1). Use of Hi-FGF-2-selective neutralizing antibodies allowed us to determine the effect of secreted human Hi-FGF-2 on the profibrotic, myofibroblast phenotype, and on cardiomyocyte hypertrophy. Our work supports the notion that human Hi-FGF-2 in the heart is a clinically relevant target, and that strategies aimed at reducing endogenous Hi-FGF-2 production or activity should be considered to prevent maladaptive remodelling associated with heart disease.

## Methods and Materials

According to Institutional policies (University of Manitoba and St.Boniface General Hospital), all surgery patients sign a consent form allowing tissue materials and fluids (removed and discarded as a normal part of surgery) to be used for research purposes. Based on this, the Research Ethics Board of the University of Manitoba waived the need for individualized informed consent by donors, and granted permission for use of human tissue and pericardial fluid from cardiac surgery patients (#H2007:004). Data were analyzed anonymously. Approval for use of 1 day old rat pups to obtain heart tissue was obtained by the Protocol Management and Review Committee of the University of Manitoba (#09-004). The pups were sacrificed by decapitation with sharp scissors. This study was carried out in strict accordance with the recommendations in the Guide for the Care and Use of Laboratory Animals by the US National Institutes of Health (NIH Publication No. 85-23, revised 1996).

### Human tissues, primary cultures, and pericardial fluid

Human atrial tissue fragments, of approximately 0.5 cm^3^ in size, were obtained from patients undergoing coronary artery bypass grafting and placed in basal medium on ice. One-half of each tissue fragment was stored in liquid nitrogen, and used to obtain tissue protein extracts. The other half was either placed in 10% formalin followed by embedding in paraffin, or used to obtain primary cultures of mobile fibroblastic cells. In the latter case, tissue was minced finely and placed in a 60 mm plastic dish with basal medium plus 10% fetal bovine serum (FBS), 100 units/ml penicillin, 100 µg/ml streptomycin (GIBCO). Cells migrating from the explants and allowed to grow for up to 2 weeks were passaged two more times before use. These cells (at P2–P3) represent human cardiac myofibroblasts (hMFs). Pericardial fluid was obtained from patients (n = 10) undergoing routine cardiac surgical procedures, through aspiration of fluid from the pericardium prior to systemic heparinization, avoiding contamination with blood. Human adult atrial or ventricular fibroblast primary cultures (NHCF-A, NHCF-V) obtained from healthy individuals were purchased from Lonza.

### Reagents and Kits

Angiotensin II (Ang II) was purchased from Sigma or Bachem. Due to variation in potency of various batches of Ang II, this reagent was used at 10^−6^ M, as this concentration gave consistent results with all batches. Losartan (Sigma), PD123319 (Tocris BioScience), and U0126 (Millipore) were used at 10^−5^ M each, while matrix metalloprotease 2 (MMP-2) inhibitor-1 (MMP2 I1, cis-9-Octadecenoyl-N-hydroxylamide, Oleoyl-N-hydroxylamide, OA-Hy; Millipore) was used at 3×10^-5^ M. Protease (PIC) and phosphatase (PPIC II and PPIC IV) inhibitor cocktails, were from Sigma and Calbiochem, respectively.

### Antibodies

Monoclonal anti-FGF-2 antibodies for western blotting (#05-118) or activity neutralization (#05-117) were purchased from EMD Millipore. Rabbit polyclonal antibodies specific for human Hi-FGF-2 were custom made (Sigma Genosys) against a sequence (GRGRGRAPERVG) present in the N-terminal extension of human Hi-FGF-2, using the same strategy as in [15]; they were affinity-purified, and used at 10-20 µg/ml. The mouse monoclonal antibodies to pro-collagen (sp1D8) or cardiac troponin T (TnT; CT3) developed by Dr. Heinz Furthmayr and Dr. Jim Jung-Ching Lin, respectively, were obtained from the Developmental Studies Hybridoma Bank developed under the auspices of the NICHD and maintained by The University of Iowa, Department of Biology, Iowa City, IA 52242. Antibodies to extra-domain fibronectin (EDA-FN), embryonic smooth muscle myosin heavy chain (SMemb), α-smooth muscle actin (α-SMA), were from Chemicon; antibodies to vimentin, N-cadherin, GAPDH, were from Abcam. Antibodies to desmin and α-actinin, were from Sigma. Antibodies for phospho ERK and total ERK were from Cell Signaling. Antibodies for Ang II Type-1 (goat; AT-1R) and Type-2 (rabbit; AT-2R) receptors, as well as plasminogen activator inhibitor-1 (PAI-1), interleukin 1-β (IL-1β), and β-tubulin were obtained from Santa Cruz Biotechnology, Inc. Secondary antibodies for Western blotting (anti-mouse and anti-rabbit immunoglobulin conjugated to horseradish peroxidase) were purchased from BioRad and used at 1∶10000 dilutions.

### Isolation of anti-human Hi-FGF-2 antibodies by affinity chromatography

Recombinant human Hi-FGF-2 (24 kDa isoform) was cross-linked to CNBr-activated Sepharose (GE Healthcare), as per manufacturer's instructions. The rabbit anti-Hi-FGF-2 antiserum (1 ml) was diluted with 9 ml binding buffer recombinant (40 mM Tris-HCl pH 8.0, 0.2 M NaCl), clarified by filtration, and incubated with human Hi-FGF-2-Sepharose (1 ml slurry) at ambient temperature, with gentle shaking for 2 hours. After extensive washing, bound immunoglobulin (IgG) was eluted with 4 M MgCl_2_ and dialyzed against 10% glycerol in binding buffer. Pure anti-Hi-FGF-2 IgG was used at 10 µg/ml for immunofluorescence, 20 µg/ml for activity neutralization, and at 1 µg per 100 µg extracted protein for immunoprecipitation.

### Expression of human FGF-2 isoforms by gene transfer

The cDNAs used to overexpress human Hi-FGF-2 (24 kDa), and rat Hi- and Lo-FGF-2, have been described in [25] and [26], respectively. The human embryonic kidney (HEK) 293 cell-line was purchased from Stratagene and transfected as in [25]. Purified recombinant His-tagged rat Hi- and Lo-FGF-2, and human Hi-FGF-2 (24 kDa) were obtained as we described[17]; briefly, corresponding cDNAs were ligated into multiple cloning sites of pET-19b vector, and transformed into competent *E.Coli*, BL21(DE3) (both from Novagen). After induction, recombinant proteins were isolated by Ni-Sepharose (GE-Healthcare) chromatography, as per manufacturer's instructions.

#### High salt elution of extracellular, cell associated FGF-2

We followed the procedure described for rat FGF-2 [19]. Briefly, after aspiration of conditioned medium, human cardiac myofibroblasts (hMFs) were gently washed with 2 ml per 100 mm dish of high salt solution (2 M NaCl in 10 mM Tris-HCl, pH 7.2), containing 0.5% BSA. High salt washes were diluted to 0.5 M NaCl with 10 mM Tris-HCl pH 7.2 supplemented with PIC, before being used to obtain the heparin-bound fraction.

### Heparin-sepharose fractions

A heparin-sepharose CL-6B slurry (100 µl) was used to obtain FGF-2-enriched fractions from pooled hMF conditioned media (60 ml/sample) or from the diluted high salt eluates (from 3×100 mm plates, pooled), as previously described[19]. Pericardial fluid (0.5 ml) was diluted to 10 mg protein/ml and made up to 0.6 M NaCl before incubation with heparin-sepharose. Conditioned media, diluted high salt eluates, and diluted pericardial fluid were maintained in the presence of PIC protease inhibitors. Heparin-sepharose bound proteins were eluted by boiling in twice-concentrated SDS/PAGE sample buffer (final concentration: 125 mmol/L Tris-HCl pH 6.8, 2% SDS, 20% glycerol, 0.010% bromophenol blue, 10% β-mercaptoethanol).

### Hypertrophy *in vitro*

Cell surface area was measured by morphometry of neonatal rat cardiomyocytes (NIH ImageJ program), at an n = 320–480 myocytes/group, stained by immunofluorescence for N-cadherin (cell periphery) and α-actinin (myofibrils) as previously described [17], [19]. Protein synthesis (^3^H-leucine incorporation) was determined as previously described[17]. Briefly, cardiomyocyte in 35 mm dishes (n = 5 dishes/group) were placed in leucine-free media that had been conditioned by either Ang II-stimulated, or non-stimulated hMFs, incubated in the presence or absence of neu-Ab^Hi-FGF-2^ (20 µg/ml) and followed by addition of ^3^H-leucine (5 µCi/well). Cells were processed for scintillation counting 24 h later.

### Immunolocalization

Immunohistochemistry of paraffin sections (4 µm) of human atrial tissue samples were used for immunohistochemical detection of human Hi-FGF-2, as described in [27]. The Vectastain ABC kit (rabbit IgG) from Vector laboratories was used. Sections were incubated with anti-human-Hi-FGF-2 rabbit polyclonal antibodies (20 µg/ml in blocking solution), followed by horseradish peroxidase (HRP)-labeled secondary antibodies and diaminobenzidine (Sigma). Sections were counterstained with haematoxylin (Vector Laboratories Inc.). Atrial tissue paraffin sections were also subjected to immunofluorescence, following de-paraffinization with successive washes in xylene, and decreasing concentrations of ethanol. Antigen unmasking of sections was achieved by immersion in 1∶100 dilution of ‘antigen unmasking solution’ (Vector Laboratories, H-3300), as per manufacturer's instructions. Tissue sections were also treated with the autofluorescence eliminator reagent as per manufacturer's instructions (Millipore). Immunofluorescence of cells in culture was done exactly as we described previously [25], [28].

### Immunoprecipitation with anti-human Hi-FGF-2 antibodies

Human embryonic cardiac fibroblasts, grown to confluency, were scraped and sonicated briefly into RIPA buffer (150 mM NaCl, 1% (v/v) NP-40, 0.25% (w/v) deoxycholate, 0.1% (w/v) SDS, 50 mM Tris-HCl pH 8.0, 1 mM EGTA, 1 mM EDTA, 1 mM Na_3_VO_4_), supplemented with protease inhibitors. For immunoprecipitation, 900 µg total extract protein were pre-absorbed with protein A-sepharose (GE Health care), and then incubated with 9 µg of either purified anti-Hi-FGF-2 IgG, or non-specific IgG. Immunocomplexes were collected with 40 µl protein A-sepharose slurry, washed extensively with RIPA buffer, and eluted by boiling into twice-concentrated SDS/PAGE sample buffer.

### Tissue/cell extraction and analysis by western blotting

Total protein extraction from cardiac tissue or cells was performed as we previously described [29]. All buffers were supplemented with protease inhibitor cocktail (PIC) as well as phosphatase inhibitor cocktails (PPIC II, IV). For FGF-2 detection by western blotting, tissue or cell lysates, at, respectively, 50–100 or 10–50 µg protein/lane, were analyzed in 15% SDS/PAGE gels. Protein concentrations were measured using BCA assay (Bicinchoninic acid; Sigma). Antigen-antibody complexes were visualized by chemiluminescence using ECL Plus (Amersham BioSciences).

### Zymography

As in [27]. Briefly, hMF conditioned medium (2 ml) was concentrated to 0.1 ml with Nanosep 10K Omega concentrators (PALL). Concentrated conditioned medium (20 µl) was mixed with sample buffer containing 20% glycerol, 4% SDS, 0.13 M Tris-HCl, pH 6.8, and resolved on a 7.5% polyacrylamide gel containing 1 mg/ml porcine gelatin (Sigma). Purified human MMP-2 (Chemicon) was used a positive control. After electrophoresis, gels were washed twice with 2.5% Triton X-100 for 30 min at room temperature to remove SDS, and placed in 50 mM Tris-HCl, 5 mM CaCl_2_, 0.2 M NaCl, pH 7.6, at 37°C for 48 h. Gels were then stained with Coomassie blue and de-stained with 40% methanol and 10% acetic acid.

### Statistical analysis

Statistical comparisons between two groups was done using t-test; for more than two groups, one-way Analysis of Variance (ANOVA) was used followed by the Tukey-Kramer multiple comparisons test, with GraphPad InStat 3.0, and GraphPad Prism. Differences among groups were defined as significant at P<0.05.

## Results

### Human FGF-2 isoform expression in human cardiac tissue

As a first step towards investigating potential relevance in the adult human heart we asked if Hi-FGF-2 isoforms were present in human myocardium. According to the literature, FGF-2, of uncertain isoform composition, is present in mammalian atria and ventricles; relative FGF-2 levels are especially high in atrial tissue [30]. We therefore used human atrial tissue in order to maximize the ability to detect all FGF-2 isoforms by western blotting analysis of unfractionated lysates. Use of unfractionated lysates from freshly obtained atrial tissue was crucial in order to prevent partial proteolytic conversion of Hi-FGF-2 isoforms to Lo-FGF-2-like proteins during handling [31], [32]. An additional argument in favor of using atrial tissue for these initial studies is that, unlike human ventricular tissue, it was possible for us to obtain a relatively large number of small tissue fragments (n = 60) from patients undergoing routine cardiac surgery and thus obtain a more comprehensive picture regarding the relative expression of Hi- versus Lo-FGF-2 in cardiac (atrial) tissue.

**Figure 1:**
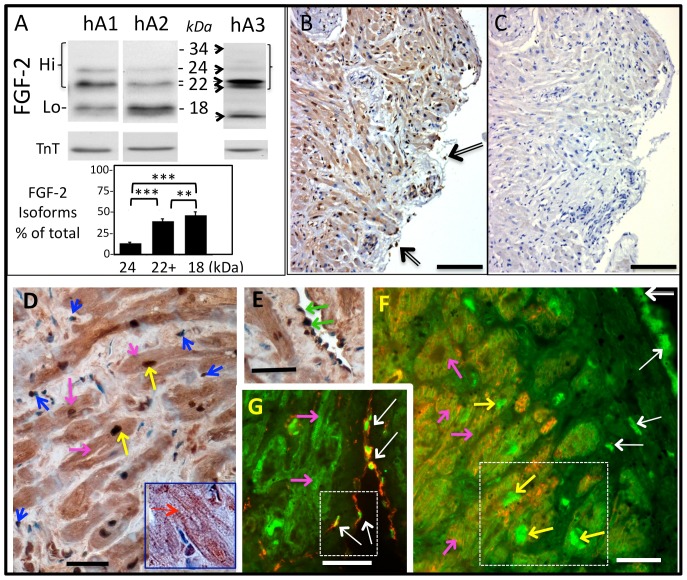
Detection of Hi-FGF-2 in human atrial tissue. **Panel (A)** shows representative western blot images of human atrial extracts (hA1, hA2, hA3, 50 µg/lane) probed for FGF-2 with an antibody detecting all FGF-2 isoforms. Expected migration of all human FGF-2 isoforms (34, 24, 22–22.5, and 18 kDa), corresponding to Hi- or Lo-FGF-2, is indicated by arrows; please note that the 34 kDa isoform is not detectable in tissue lysates. Western blots were also probed for cardiac troponin T (TnT) to verify equivalent loading of lanes. Samples hA1, hA2 were analyzed in small (8.3×5.5 cm^2^) 15% polyacrylamide gels, while hA3 was analyzed in a large (16×11.5 cm^2^) 15% polyacrylamide gel. The included graph shows percentage of each isoform over total FGF-2, where, n = 45; comparisons between groups are indicated by brackets, where *** and ** denote P<0.001, and P<0.01, respectively. **Panels (B) and (C)** show images from patient-derived serial atrial sections, subjected to (B) incubation with purified anti-Hi-FGF-2 antibodies followed by immunohistochemical visualization of antigen-antibody complexes (brown color) as well as nuclear staining (hematoxylin, blue), and (C) similar procedures as in B but without the anti-Hi-FGF-2 antibodies. Incubation with anti-Hi-FGF-2 antibody elicits extensive immunostaining, in what appears to be nuclear as well as cytosolic sites in cardiomyocytes; staining of non-cardiomyocytes located at the epicardium is indicated by arrows. **Panels (D) and (E)** are close-up images from human atrial tissue sections stained as in (B), showing cellular and subcellular distribution of Hi-FGF-2. **Panels (G) and (F)** show human atrial sections subjected to double-immunofluorescence staining for Hi-FGF-2 (green), and either vimentin (G, red), or desmin (F, red). In all images, yellow or pink arrows point, respectively, to nuclear or cytosolic sites within cardiomyocytes. Blue arrows in (D) point to small connective tissue cells, likely fibroblasts. Green arrows in (E) point to endothelial cells, lining a vessel. White arrows in (G) and (F) point to non-myocytes, found at or near the epicardial region. These cells are positive for vimentin, but not desmin. In (F), co-staining with desmin confirms presence of Hi-FGF-2 in atrial cardiomyocytes. Sizing bars in (B) or (D,E,F,G) correspond to 250 or 100 µM, respectively. Insets within panels G and F are shown in larger magnification in Fig S2.

Tissue lysates were analyzed for FGF-2 isoforms by western blotting and densitometry. Representative western blot images labeled as hA1 and hA2, derived from small 15% polyacrylamide gels, are shown in Fig. 1A. Immunoreactive bands were detected at 18 kDa (Lo-FGF-2), as well as at 22 and 24 kDa (Hi-FGF-2). The 22 kDa band resolved into a doublet (22 and 22.5 kDa Hi-FGF-2 isoforms) when samples were analyzed in large 15% polyacrylamide gels, and a typical pattern is shown in Fig. 1A, labeled as hA3. The 18, 22–22.5 and 24 kDa bands compose, respectively, 47%, 39% and 14% of total atrial tissue-derived FGF-2 (Fig. 1A). Hi-FGF-2 constituted 53% (±20 SD, n = 45) of total tissue FGF-2. Using a recombinant FGF-2 standard curve and densitometry, we estimated that human atrial extracts contained 1.78 (±0.09, SEM) pg total FGF-2 per µg of extracted protein. Fig. S1 shows a western blot of recombinant FGF-2 (12.5–200 pg/lane) side-by side with selected representative human atrial extract samples (50 µg/lane), to illustrate that the intensity of immunoreactive signals in human tissue samples was within range shown by the recombinant FGF-2 samples.

To determine cellular and subcellular distribution of Hi-FGF-2 in human atrial tissue we used immunocytochemistry with affinity-purified anti-human Hi-FGF-2 antibodies. Fig.1 (B, C) shows atrial serial sections incubated in the presence (Fig.1B) or absence (Fig.1C) of anti-Hi-FGF-2 antibodies. Staining of antigen-antibody complexes elicited brown color, while counter-staining with hematoxylin (both B, C) elicited blue color, visualizing nuclei. Relatively extensive brown staining was seen only in (B) consistent with a specific reaction for Hi-FGF-2. Cells that were present near the epicardium and stained positive for Hi-FGF-2 are indicated by arrows in Fig.1B. Close-up images of the anti-Hi-FGF-2 staining patterns are shown in Fig.1 (D, E). Hi-FGF-2 was localized in nuclei (yellow arrows) and cytosol (pink arrows) of atrial cardiomyocytes. Cardiomyocytes were recognized by their relatively large size, long cylindrical shape, prevalence in tissue, and presence of striations (see inset in Fig.1D). Hi-FGF-2 was also localized in non-cardiomyocytes, including the endothelial blood vessel lining (green arrows in E), and small connective tissue cells with fibroblastic appearance (blue arrows in D). Double-immunofluorescence staining of human atrial tissue sections for Hi-FGF-2 (green) and the muscle cytoskeletal protein desmin (red), Fig.1F, confirmed presence of Hi-FGF-2 within cardiomyocytes, in both nuclear and cytosolic sites. Simultaneous fluorescence staining for nuclei confirmed nuclear localization of Hi-FGF-2 in atrial cardiomyocytes; Fig.S2 (A, B). As in Fig.1B, Hi-FGF-2 was localized in cells near the epicardium that were also positive for the mesenchymal/fibroblastic marker vimentin (Fig.1G). A higher magnification image of these cells is included in Fig.S2C. Tissue sections were obtained from 5 patients and all displayed similar patterns of Hi-FGF-2 localization as shown in Fig.1B (patient 1), and Fig.1 (D, E; patient 2). Commercially available atrial sections from a healthy individual were also examined, and again displayed an immunostaining pattern similar to that shown in Fig.1 (B, D, E) and Fig.S2D. Taken together, experimental data included in Fig.1 show that Hi-FGF-2 is indeed present in human atrial tissue, constituting, on average, about half of total FGF-2, and that it is associated with cardiomyocytes as well as non-myocytes including connective tissue cells. Cardiomyocytes and extracellular space constitute 45% and 49% of human atrial volume, with non-cardiomyocytes such as endothelial cells and connective tissue cells making up the rest[33]. The bulk of tissue lysate-extracted FGF-2 therefore is likely to be dominated by cardiomyocyte-FGF-2, as well as extracellular matrix-bound FGF-2, secreted or released by cardiac cells.

Characterization of the anti-human Hi-FGF-2 antibodies used here is included in Figs.S3 and S4. Firstly we asked if anti-Hi-FGF-2 could detect human Hi-FGF-2 in cells *in situ*, by immunofluorescence. We used transient gene transfer to introduce cDNAs modified to express human Hi-FGF-2 only, or Lo-FGF-2 only, in a transformed cell line with relatively low levels of endogenous FGF-2, the human embryonic kidney (HEK293) cells, as we have done in previous studies[25]. We then probed, by dual immunofluorescence, for Hi- or total FGF-2 expression, using affinity purified anti-human Hi-FGF-2 antibodies, or commercially available monoclonal antibodies recognizing all FGF-2 isoforms. The latter served to document FGF-2 (Hi- or Lo-) overexpression. As seen in Fig.S3, anti-Hi-FGF-2 antibodies detected overexpressing cells only in cultures transfected with the cDNA for human Hi-FGF-2. By western blotting of denatured proteins we found that anti-Hi-FGF-2 could only detect purified recombinant human Hi-FGF-2, but not recombinant rat Hi-FGF-2 or rat Lo-FGF-2 (Fig.S4A). In a third experiment we asked if the anti-human Hi-FGF-2 antibodies could interact with and immunoprecipitate native human Hi-FGF-2, but not Lo-FGF-2, from non-transfected cell extracts. We used extracts from commercially available primary human embryonic cardiac cells, and found that anti-Hi-FGF-2 could indeed immunoprecipitate the 22-24 kDa human Hi-FGF-2, but not Lo-FGF-2 from these extracts (Fig.S4C).

### FGF-2 Isoform Expession in Human Atria-Derived Myofibroblasts

Fibroblasts and myofibroblasts are considered to be important sources of secreted FGF-2 in various tissues. We have shown that in the rat, heart ventricle-derived myofibroblasts express, and secrete, predominantly Hi-FGF-2 [19]. There is as yet no information as to the relative FGF-2 isoform expression and/or secretion by adult human cardiac myofibroblasts, atrial or ventricular. To address this issue we used patient-derived atrial explants to isolate migratory, proliferative cells that were identified as ‘activated fibroblasts’, or myofibroblasts (hMFs). Identification was based on fibroblastic morphology, presence of stress-fibers, and expression of fibroblast and/or myofibroblastic marker proteins including co-expression of vimentin and α-smooth muscle actin (α-SMA), expression of embryonic smooth muscle myosin (SMemb) as well as expression of extracellular matrix proteins, such as collagen (in its procollagen form), and extra domain A (EDA)-Fibronectin [2], [34], shown in Fig. S5.

**Figure 2:**
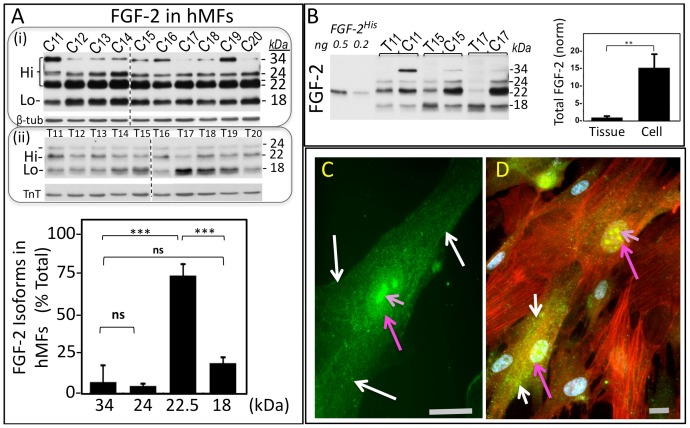
Detection of Hi-FGF-2 in human atrial myofibroblasts. **Panel (A)** shows two sets of western blots analyzing FGF-2 isoforms. The first set, (i), is a composite of two blots (separated by a broken line) and analyzes FGF-2 isoforms in hMF lysates (20 µg/lane), from atrial myofibroblast primary cultures obtained from 10 patients (patients 11–20), and correspondingly labeled as C11–20. The second set, (ii), also a composite of two blots separated by a broken line, analyzes FGF-2 isoforms in atrial tissue lysates from patients 11–20, and labelled T11–20 (50 µg/lane). The hMF blots or tissue blots were also probed for, respectively, β-tubulin (β-tub), or Troponin-T (TnT), as indicated. Following densitometry of the hMF blots, the % contribution of each FGF-2 isoform to the total FGF-2 signal was determined for each individual lane, and cumulative results (mean±SD) are included in graph form (n = 10). **In Panel (B)**, a western blot shows FGF-2 signals from 0.5 and 0.2 ng/lane of recombinant histidine tagged Lo-FGF-2 (FGF-2*^His^*), atrial tissue lysates (T11, T15 and T17, loaded at 50 µg/lane), side by side with FGF-2 signals from lysates obtained from corresponding primary hMF cultures (C11, C15 and C17, loaded at 10 µg/lane). The graph shows comparisons between tissue and cell lysates for their relative total FGF-2 content, assessed by densitometry as optical density (O.D.) units (n = 3). Measurements corresponding to cell FGF-2 were multiplied by 5, to correct for the 5-fold difference in total protein loading. In both panels, comparisons between groups are indicated by brackets, where P>0.05 is marked as ns, while P<0.001, 0.01, are marked as ***, or **, respectively. **Panels C and D** show immunofluorescence images of hMFs stained for, (C), Hi-FGF-2 (green), as well as, (D), alpha smooth muscle actin (red) and nuclei (blue). White arrows point to cytosolic Hi-FGF-2; pink and pale- pink arrows arrows point to nuclear and nucleolar Hi-FGF-2, respectively. Grey sizing bars correspond to 20 µm.

Use of readily available patient-derived atrial tissue allowed us to have an ongoing tissue source for preparation of primary cultures, providing sufficient cellular material at early passages (P2–P4) for completion of our studies. Having access to both the originating tissue and hMFs from that tissue, furthermore, enabled us to compare FGF-2 content and isoform composition between tissue-, and tissue-derived cell lysates, to examine the possibility that the isolated cells might carry a tissue ‘signature’ regarding FGF-2 expression. Fig. 2A shows FGF-2 isoform detection in primary cultures, arbitrarily labeled as C11–20, derived from 10 different patients (patients 11–20); also included in the figure is a western blot showing the FGF-2 signal from lysates of the originating tissues (T11–20). The anti-FGF-2 signal in hMF lysates represents cell-associated (intracellular as well as cell surface-bound externalized protein) FGF-2 isoforms. All hMF cultures expressed all five human FGF-2 isoforms [35] at 18, 22+22.5, 24, 34 kDa, Fig.2A. The 34 kDa FGF-2 was not detectable in the originating tissue lysates (Fig.1 and Fig.2). All hMF cultures accumulated predominantly Hi-FGF-2 (from 76–91% of the total), with a mean value of 83±4 (SD)% of total FGF-2; n = 10. In comparison, the Hi-FGF-2 percentage in the ten originating tissues used for this experiment ranged from 25–76% of total FGF-2, with a mean value of 55±14 (SD)%, n = 10. Please note that this determination was not significantly different to the value obtained from the first group of samples (n = 45) shown in Fig.1. In hMFs, the 22–22.5 kDa FGF-2 (the bulk of Hi-FGF-2 isoforms) was significantly (4-fold) higher than the 18 kDa Lo-FGF-2. The relative contribution of the 34 kDa FGF-2 displayed a high degree of variability between cultures compared to the other FGF-2 isoforms, with cultures C11 and C19 presenting a relatively strong signal. Regardless of whether the originating tissue contained predominantly Hi- or Lo-FGF-2, primary myofibroblast cultures from that tissue accumulated predominantly Hi-FGF-2, thus there was no correlation between tissue and hMF FGF-2 isoform composition (correlation coefficient, Pearson r = 0.23). This is illustrated clearly in Fig.2B, where selected tissues lysates (T11, T15, T17), are analyzed side by side with lysates from hMFs derived from these tissues (C11, C15, C17) and compared for their relative FGF-2 content and isoform composition. To obtain anti-FGF-2 signals of near-equivalent intensity it was necessary to load 5-fold more tissue- (compared to cell-) lysate protein/lane. Hi-FGF-2 in lysates from T11,T15 and T17 was at 76%, 43% and 25% of the total FGF-2 signal while that for corresponding hMFs was at 91%, 81% and 83% of the total. Relative levels of hMF-associated FGF-2 were about 15-fold higher than in tissue lysates, as shown in Fig.2B. There was no correlation regarding total FGF-2 content between tissue- and hMF- lysates (correlation coefficient, Pearson r = 0.24). Total FGF-2 content as well as isoform composition in tissue lysates is likely to be dominated by cardiomyocyte-associated FGF-2, with smaller contributions from fibroblasts and other non-myocytes, and influenced by patient pathology and drug treatments. The FGF-2 content and isoform composition in our primary cultures reflects the properties of a hyper-synthetic, hyper-contractile and hyper-secretory myofibroblast state, and supports the notion that the conversion of endogenous cells to such a myofibroblast phenotype *in vivo* would result in potent local production of Hi-FGF-2.

Immunofluorescence staining was used to examine the subcellular localization of Hi-FGF-2 in hMFs: staining with anti-Hi-FGF-2 antibodies indicated that Hi-FGF-2 localized not only to the nucleus and nucleoli, as expected from previous reports [12], [36], but also in the cytosol. The cytosolic anti-Hi-FGF-2 staining presented a granular and thread-like appearance; Fig.2 (C, D).

We asked whether predominant accumulation of Hi-FGF-2 isoforms was a common characteristic between hMFs from various sources. FGF-2 isoform composition was determined in myofibroblasts from healthy human atrial tissue (adult), healthy human ventricular tissue (adult); and human embryonic ventricular tissue. Rat ventricular myofibroblasts were also analyzed for comparison.

All of these different hMFs expressed Hi-FGF-2, including the 22-22.5, 24 and 34 kDa Hi-FGF-2 isoforms, comprising over 80% of total cell-associated FGF-2; Fig.S6A. Rat MFs expressed, as expected from our previous report[19], the 21.5, 20 and 18 kDa FGF-2 isoforms[37]. Relative total FGF-2 levels in human adult ventricular or atrial hMFs were significantly (4-fold, P<0.05, n = 3) higher than those in adult rat ventricular MFs. Overall the FGF-2 isoform composition was similar between adult patient-derived atrial fibroblasts, adult normal heart-derived atrial or ventricular hMFs, as well as embryonic ventricular hMFs. Thus regardless of atrial versus ventricular origin, or adult versus embryonic stage, cardiac tissue-derived myofibroblasts accumulate predominantly Hi-FGF-2. It should be noted that atrial fibroblasts have been shown to exhibit a hightened reactivity to various growth factors, and an enhanced profibrotic potential compared to their ventricular counterparts[38]. Although our studies show no differences regarding the ability to express and secrete Hi-FGF-2 between human atria and ventricular myofibroblasts, a systematic study would be required to determine whether there are differences in the regulation of Hi-FGF-2 expression and secretion between these two cellular populations.

In addition to myofibroblasts, endothelial cells produce growth factors contributing to tissue remodelling[39]; sublethal oxidative damage of endothelial cells is reported to result in the release of both Hi- and Lo-FGF-2 isoforms to the extracellular space[31]. We compared cell-associated Hi-, and Lo-FGF-2 levels between human primary endothelial cells (lymphatic and aortic) and atrial hMFs. Atrial hMFs accumulated over 20-fold more FGF-2 (both Hi- and Lo- isoforms) compared to either lymphatic or aortic endothelial cells; the 34 kDa Hi-FGF-2, furthermore was not detectable in the endothelial cells tested; Fig. S6B. Our data suggest that, (a), compared to endothelial cells, hMFs are likely to be a more significant source of Hi-FGF-2 in cardiac interstitium, and (b), accumulation of high levels of Hi-FGF-2 by human myofibroblasts is not likely to be an artifact caused by conditions *in vitro*, but rather it represents a genuine cell type (myofibroblast)-related property.

### Regulation of human FGF-2 isoform production by Angiotensin II, *in vitro*

Several chronic cardiovascular diseases characterized by myofibroblast-induced maladaptive remodelling, including hypertension, coronary heart disease, atherosclerosis, heart failure, fibrosis are linked to elevated levels of Angiotensin II (Ang II), and activation of Ang II receptors[22], [24]. We next investigated the effect of the Ang II on hMF Hi-FGF-2 accumulation and secretion, *in vitro*.

**Figure 3:**
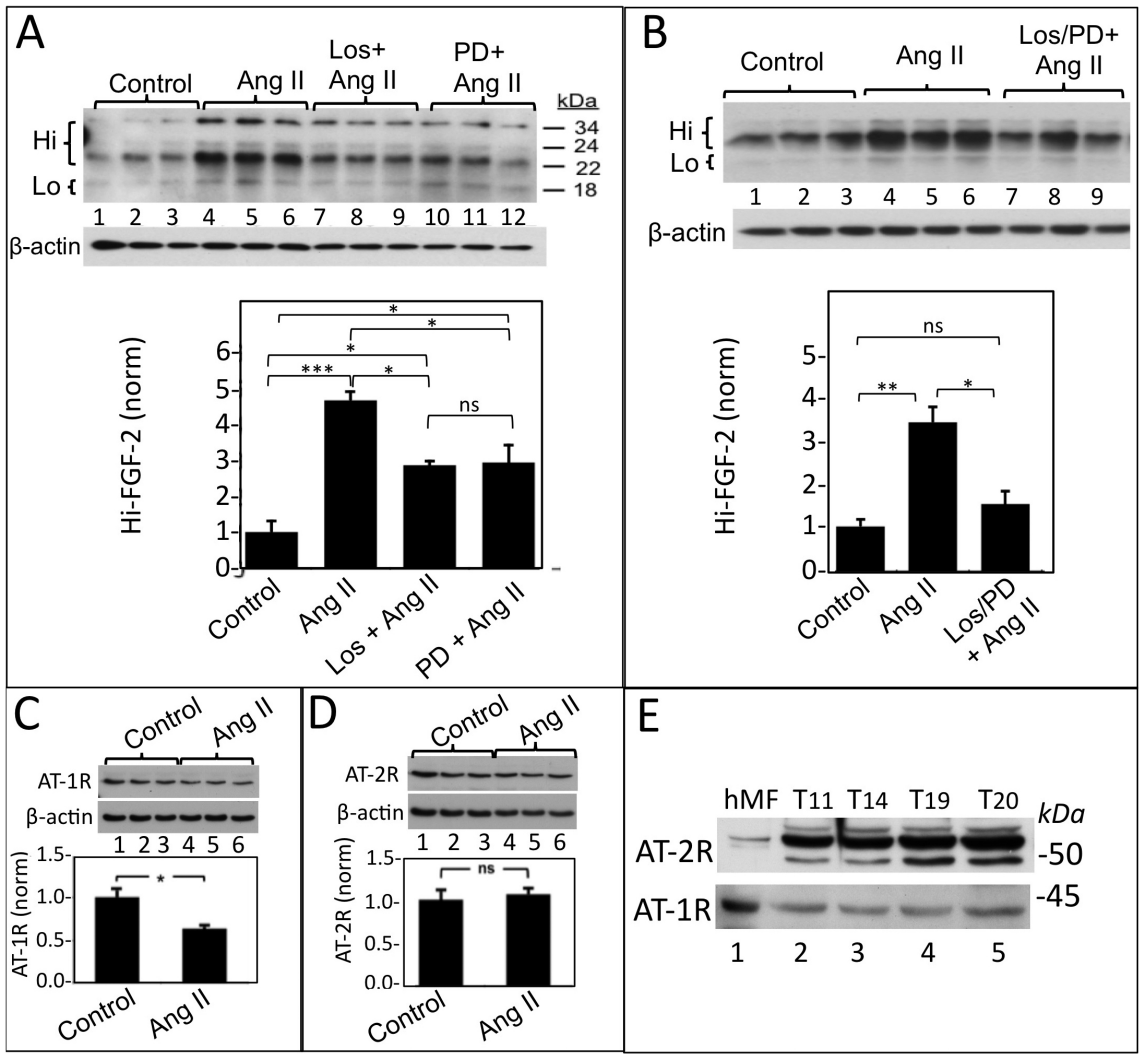
Angiotensin II promotes upregulation of cell-associated human Hi-FGF-2 via AT-1R and AT-2R. **Panel A**: western blot, and corresponding cumulative data, showing the effect of Ang II on Hi-FGF-2 accumulation by hMFs, in the absence or presence of either losartan (AT-1R inhibitor) or PD123319 (AT-2R inhibitor). Lanes 1–3; 4–6; 7–9; 10–12 correspond to lysates from, respectively, untreated (Control)-;Ang II-stimulated-; Ang II stimulated in the presence of losartan; and Ang II-stimulated in the presence of PD123319- hMFs. Ang II promotes Hi-FGF-2 upregulation which is significantly decreased by either losartan or PD123319. **Panel B**: western blot and cumulative densitometry data showing the effect of Ang II on Hi-FGF-2 accumulation in the absence or presence of simultaneous inhibition of both AT-1R and AT-2R. Lanes 1–3; 4–6; 7–9 correspond to lysates from, respectively, untreated (Control)-;Ang II-stimulated-; Ang II stimulated in the presence of both losartan and PD123319- hMFs. Relative levels of Hi-FGF-2 in the presence of both AT-1R and AT-2R inhibitors are not significantly different to those of unstimulated cells. **Panels C and D**. Western blots showing expression, respectively, of AT-1R or AT-2R by hMFs, and relative levels of these receptors after 24 h stimulation with Ang II. After 24 hour stimulation, levels of AT-1R, but not AT-R2, decrease compared to unstimulated cells. Signal for β-actin is also shown in A-D, serving as loading control. **E**. Densitometry data showing the effect of Ang II receptor inhibitors on baseline Hi-FGF-2 accumulation by hMFs in the abcence of stimulation by added Ang II. Incubation of unstimulated hMFs with losartan (but not PD123319) significantly decreased baseline Hi-FGF-2 levels. Sample size n = 3 (all graphs); *, **, *** indicates P<0.05, <0.01, <0.001, respectively; and ns denotes non-significance difference at P>0.05.

Fig. 3A shows that stimulation with Ang II elicited a significant increase in cell-associated 22–34 kDa Hi-FGF-2. The Ang II-induced increase in Hi-FGF-2 was significantly reduced in the presence of either losartan (AT-1R inhibitor), or PD123319 (AT-2R inhibitor), by 50% and 46%, respectively. In cells stimulated with Ang II in the presence of either inhibitor alone, Hi-FGF-2 levels remained significantly higher than those of the non-stimulated controls; Fig. 3A. Hi-FGF-2 levels in cells stimulated with Ang II in the presence of both inhibitors were not significantly different than those of unstimulated cells, Fig. 3B, suggesting that AT-1R and AT-2R may promote Hi-FGF-2 accumulation in an additive manner. Western blot analysis confirmed that both AT-1R and AT-2R were expressed by hMFs and that stimulation with Ang II for 24 h resulted in down-regulation of the AT-1R but not AT-2R. AT-1R and AT-2R were detected, by western blotting, in patient atrial tissue lysates (Fig.3E), suggesting that these receptors may be mediating human FGF-2 and Hi-FGF-2 production *in vivo*.

**Figure 4:**
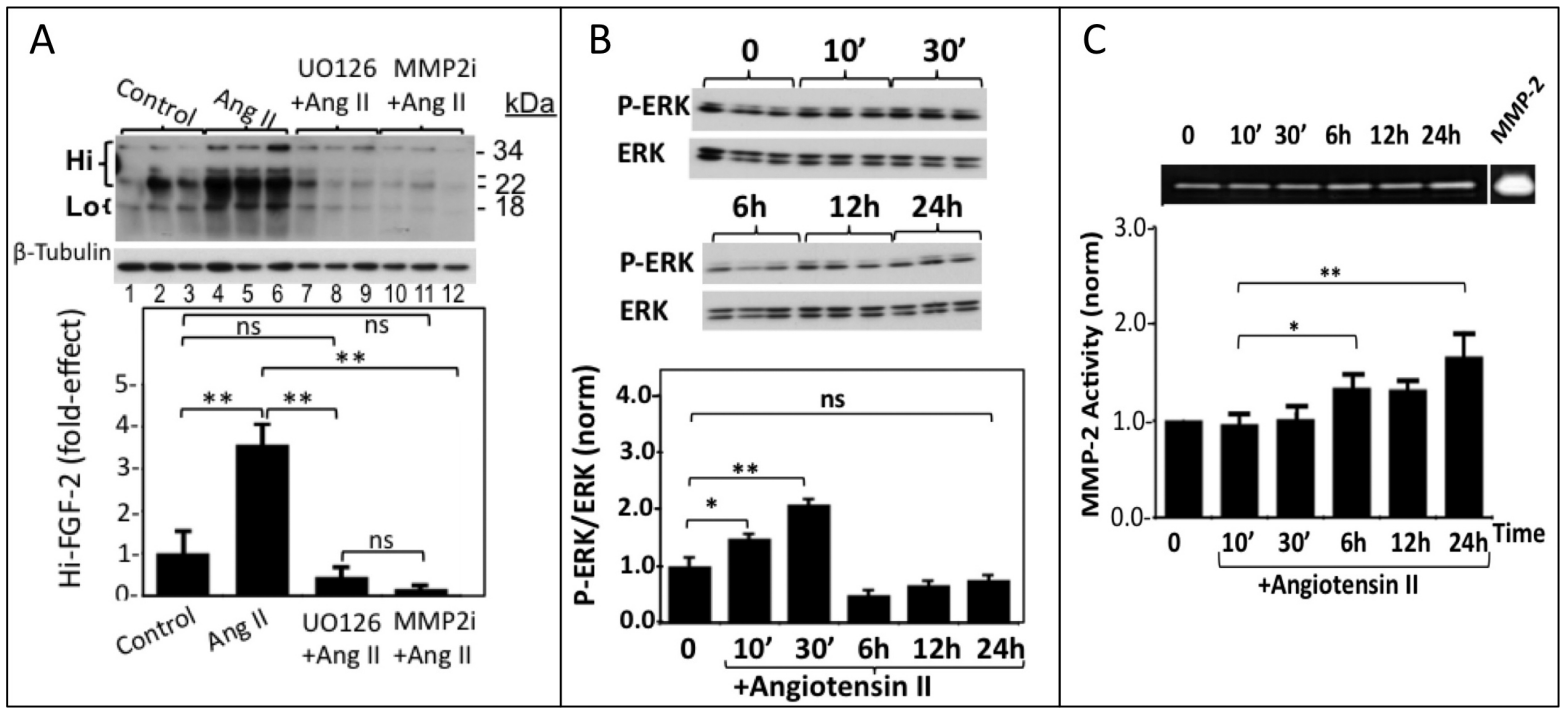
ERK and MMP-2 activities mediate the Ang II-induced Hi-FGF-2 upregulation in hMFs. **Panel A.** Western blot and corresponding cumulative data showing the effect of an ERK inhibitor (U0126), or MMP-2 inhibitor (MMP2i) on the Ang II induced Hi-FGF-2 upregulation. Signal for β-tubulin is also shown, serving as loading control. **Panel B.** Western blots and corresponding cumulative data showing the effect of Ang II administration on phospho-(P)-ERK and total ERK, after 10–30 minutes and 6–24 hours of stimulation as indicated. The graph shows cumulative data (n = 3) of the ratio between P-ERK/ERK over time (10–30 min, 6–24 hours), in response to Ang II. Minutes and hours are indicated as ‘ and h. **Panel (C)** Representative zymogram of MMP-2 activity in hMFs, including a positive control band (MMP-2), and corresponding cumulative data, showing relative MMP-2 activity in response to Ang II, over time (10–30 min, 6–24 hours), as indicated. For all graphs, brackets show comparisons between groups; *, **, ***, and ns correspond to P<0.05, P<0.01, P<0.001, and P>0.005, respectively.

Expression of the FGF-2 gene, as well as total FGF-2 protein accumulation, are regulated by ERK (extracellular signal activated kinase) [7], as well as matrix metalloproteinase (MMP-2) activities [40], although there is no information about the role of these signals on Hi-FGF-2 accumulation. We asked if the Ang II-induced upregulation of cell-associated Hi-FGF-2 required the activity of the ERK pathway, and/or MMP-2. Inhibition of the ERK activating pathway (with UO126), or MMP-2 activity (with MMP-2 Inhibitor) prevented the Ang II-induced increase in 22–34 kDa Hi-FGF-2 (Fig.4A). The ability of Ang II to stimulate ERK activity in hMFs was confirmed, and shown in Fig. 4B. Ang II increased ERK activity at 10 and 30 min from stimulation, without affecting total ERK levels. Ang II also elicited a small increase in MMP-2 activity, becoming significant at 6 and 24 hours after stimulation (Fig. 4C).

**Figure 5:**
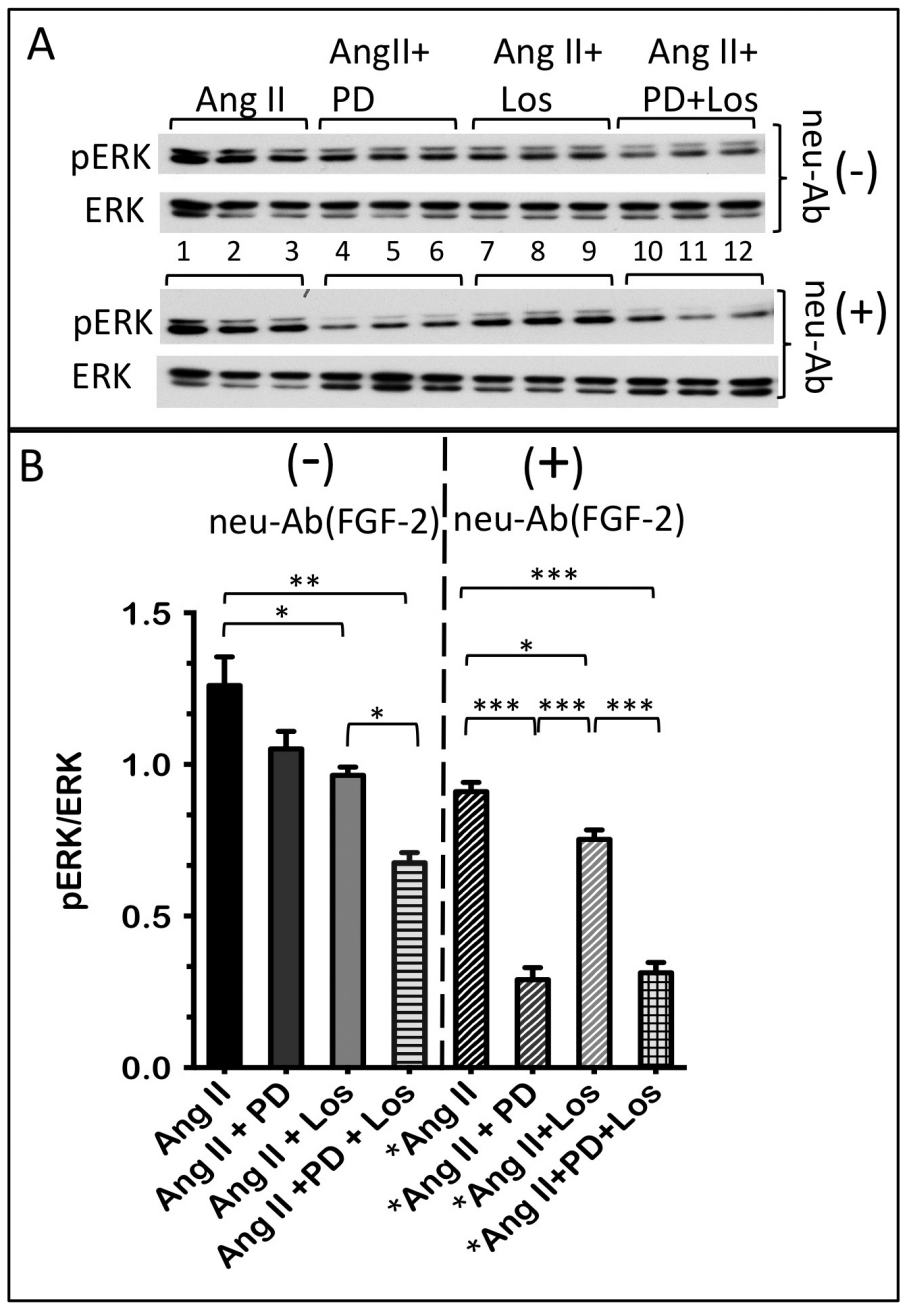
Both AT-1R and AT-2R mediate the Ang II-induced ERK activation in hMFs. Panel A shows western blot of activated (phosphorylated) pERK, and total ERK, in hMFs stimulated for 30 minutes with with Ang II (lanes 1,2,3), Ang II + PD123319 (lanes 4,5,6), Ang II + Losartan (lanes 7,8,9), and Ang II +PD123319 +Losartan (lanes 10,11,12), in the absence (-) or presence (+) of neutralizing anti-FGF-2 antibodies (neu-Ab^FGF-2^), as indicated. Please note that the western blot for pERK in the groups incubated with neu-Ab^FGF-2^ is not directly comparable to the western blot for pERK in the groups incubated in the absence of neu-Ab^FGF-2^ (different exposures). Panel B shows pERK/ERK ratios in the groups shown in panel A. Brackets show statistically significant differences between groups, where *, **, ***, correspond to P<0.05, 0.01, and 0.001, respectively.

To address Ang II receptor involvement in Ang II-induced ERK activation, hMFs were stimulated with Ang II for 30 minutes in the absence or presence of AT-1R and/or AT-2R inhibitors. Losartan elicited a 23% decrease in ERK activity, measured as the ratio of pERK/ERK, Fig.5. PD123319 did not exert a statistically significant effect although a trend towards decreasing ERK activity (by about 17%) was observed. Inhibition of ERK activity in the presence of both inhibitors, by 42%, was significantly lower than that by losartan alone, suggesting an additive effect of AT-1R and AT-2R mediated pathways to ERK activation. The same experiment was conducted in the presence of neutralizing antibodies (neu-Ab^FGF-2^, 20 µg/ml), to block the effect of extracellular-acting FGF-2 on ERK activity. ERK activity in hMFs stimulated with Ang II in the presence of neu-Ab^FGF-2^ is decreased to about 62% of that in the absence of neutralizing antibodies. In the presence of neu-Ab^FGF-2^, PD123319 elicited a very significant decrease in Ang II-induced ERK activity, by 70% of the value in the absence of the inhibitor. Losartan also decreased ERK activity, by 17%. In the presence of both inhibitors, ERK activity was decreased to the same extent as with PD123319 alone. Taken together these experiments indicate that both AT-1R and AT-2R are mediating ERK activation in response to Ang II stimulation, and suggest that the AT-1R-mediated activation of ERK may be partially dependent on the contribution of extracellular-acting FGF-2.

Levels of MMP activity in conditioned media of hMFs bstimulated with Ang II for 30 min remained unchanged in the presence of either Losartan, or PD123319, or neu-Ab^FGF-2^; (Fig. S7).

### Human Hi-FGF-2 export

**Figure 6:**
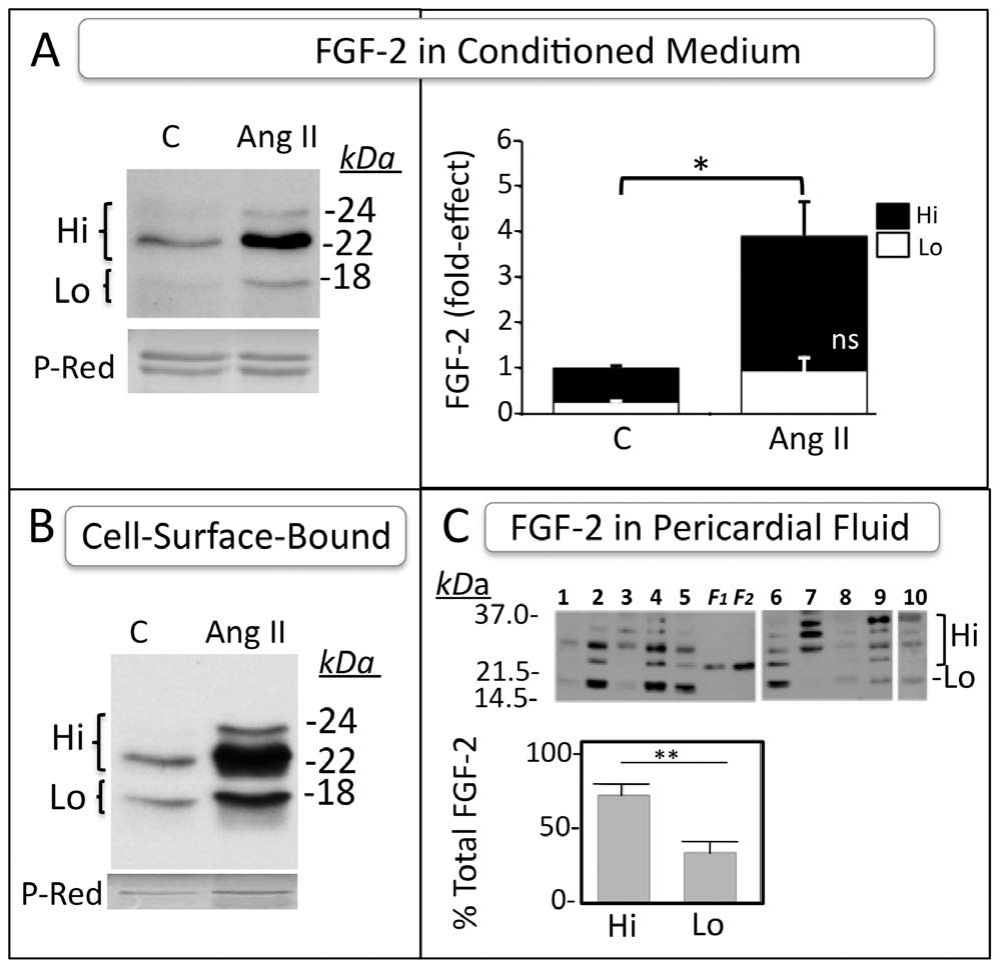
Detection of Hi-FGF-2 in the extracellular environment *in vitro* and *in vivo*. **Panel (A)**. Representative western blot images of FGF-2 detection in conditioned medium from unstimulated or Ang II-stimulated hMFs. Each lane contains the heparin-sepharose-bound fraction from 60 ml of pooled conditioned medium. **Panel (B)**. Representative western blots for FGF-2 “eluted” from the cell surface with a high salt wash, and concentrated by binding to heparin-sepharose. Each lane contains the heparin-bound fraction from a 10 ml wash (5×100 near-confluent plates). Ponceau S Red (P-Red) staining of unidentified protein band(s) is also shown, indicative of equivalent loading. Experiments shown in A and B were repeated 2 more times, with similar results. **Panel (C)**. Western blot image, and corresponding quantitative data of FGF-2 isoforms present in human pericardial fluid (n = 10). Lanes 1-5 (gel 1) and 6-10 (gel 2) contain the heparin-sepharose-bound fraction from 0.5 ml pericardial fluid of individual patients. Lanes marked as F1, F2 contain recombinant Lo-FGF-2 (histidine-tagged) loaded at 0.25 and 0.5 ng/lane respectively. Sample 10 was deliberately overexposed to increase visibility of bands. Recombinant FGF-2, used as standard, was included in the second gel as well (not shown here). The graph shows percent contribution of Hi- or Lo-FGF-2 isoforms to the total FGF-2 signal (mean ± SEM). In all panels, brackets show comparisons between groups; * and ** correspond to P<0.05 and P<0.01, respectively.

To consider human Hi-FGF-2 as a potential trigger of paracrine, autocrine signaling it is important to examine if this protein can be externalized/secreted, if it is accumulating in the extracellular environment *in vitro* and/or *in vivo*, and if extracellular levels become upregulated by pathology-associated stimuli such as Ang II. We looked for presence of FGF-2 isoforms in hMF conditioned medium, as well as in cell ‘eluates’ containing proteins bound to the cell surface and the extracellular matrix. As seen in Fig. 6A, cells exported Hi-FGF-2, detectable in both the conditioned medium as well as in the cell-surface/matrix- bound fraction. Lo-FGF-2 was below threshold of detection in conditioned medium, but was detectable in the cell-surface associated fraction (Fig. 6B). Very similar findings were obtained when using human ventricle-derived myofibroblasts, as shown in Fig. S8. Thus both atria- or ventricle-derived hMFs export FGF-2, consisting predominantly of Hi-FGF-2.

Stimulation of atria-derived hMFs with Ang II elicited a significant, 4-fold increase in Hi-FGF-2 present in conditioned medium, representing 75% of total FGF-2 (Fig. 6A). A clear increase in exported FGF-2 was seen in the cell-associated fraction, again composed predominantly of Hi-FGF-2 (Fig. 6B). The 22-22.5 kDa FGF-2 isoform was the predominant Hi-FGF-2 isoform present in the exported FGF-2 pools. The experiments shown in Fig.6 do not specifically address whether Ang II actively promotes the FGF-2/Hi-FGF-2 export process in hMFs, although they do suggest a positive correlation between levels of cell-associated FGF-2 and FGF-2 detected in both conditioned medium and in association with cell surface/matrix. The export of FGF-2 and rat Hi-FGF-2 has been found to require caspase-1 activity [19], [41], and a similar mechanism is likely mediating export of human Hi-FGF-2. In the absence of stimulation with added Ang II, baseline levels of myofibroblast-exported Hi-FGF-2 are likely a reflection of baseline levels of caspase-1 activity, as well as baseline levels of activation of the Ang II-related signaling pathway.

Detection of Hi-FGF-2 in human atrial tissue, in combination with our *in vitro* experiments showing that human Hi-FGF-2 is exported to the extracellular environment, raised the possibility that Hi-FGF-2 may be present in biological fluids *in vivo*. We tested for presence of FGF-2 isoforms in human pericardial fluid, in a pilot study including samples from 10 cardiac surgery patients. As seen in Fig. 6C, Hi-FGF-2 (22–34 kDa) as well as Lo-FGF-2 were detected in the pericardial fluid of all patients. On average, Hi-FGF-2 comprised 68 (±25 SD) % of total FGF-2, significantly higher than Lo-FGF-2; Fig. 6C. Using an FGF-2 standard curve we estimated the average total FGF-2 concentration in pericardial fluid to be at 578 pg/ml (±354 SD), which is within the 260–770 pg/ml range reported by others[42].

### Biological activity of human Hi-FGF-2

Presence in the extracellular environment raises expectations that human Hi-FGF-2 can exert autocrine or paracrine biological effects, by activating “outside-in” signal transduction. Maladaptive tissue remodelling in chronic heart disease includes paracrine stimulation of cardiomyocyte hypertrophy, conversion of non-myocytes to a myofibroblast phenotype promoting fibrosis[2], and an increased innate inflammation response[3]. We therefore examined the effects of extracellular-acting human Hi-FGF-2 on the myofibroblast phenotype, on expression of proteins linked to inflammation, and on cardiomyocyte hypertrophy.

**Figure 7:**
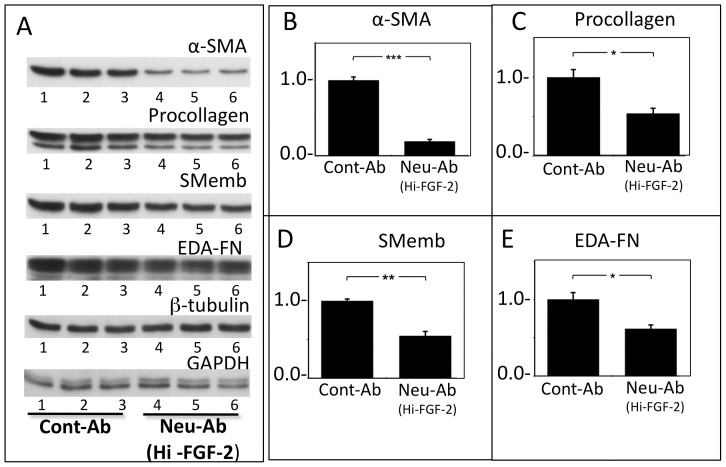
Selective neutralization of extracellular human Hi-FGF-2 attenuates expression of pro-fibrotic proteins. **Panel A**. Western blots showing the effect of incubation with either control antibodies (Cont-Ab, 20 µg/ml, lanes 1,2,3), or anti-Hi-FGF-2 antibodies (Neu-Ab^Hi-FGF-2^, 20 µg/ml, lanes 4,5,6) on the accumulation of α-SMA, procollagen, SMemb, EDA-Fibronectin (EDA-FN), β-tubulin, and GAPDH, by hMFs, as indicated. **Panels B**,**C**,**D** and **E** show corresponding quantitative (densitometry) data for α-SMA, procollagen, SMemb, EDA-Fibronectin (EDA-FN), as indicated (±SEM). Incubation with Neu-Ab^Hi-FGF-2^ significantly decreased expression of α-SMA, procollagen, SMemb and EDA-Fibronectin, without having any effect on GAPDH or β-tubulin. Brackets show comparisons between groups, where *, **, *** correspond to P<0.05, <0.01, <0.001; n = 3/group.

Cells (hMFs) were incubated with antibodies specific for Hi-FGF-2 (Neu-Ab^Hi-FGF-2^), aimed at neutralizing the effects of endogenously produced and externalized Hi-FGF-2. Control cultures were incubated with non-specific immunoglobulin. As shown in Fig. 7, hMFS incubated with Neu-Ab^Hi-FGF-2^ displayed, compared to controls, significantly decreased levels of α-SMA, EDA-Fibronectin, SMemb, and procollagen, all protein markers of a myofibroblast pro-fibrotic phenotype. Treatment with Neu-Ab^Hi-FGF-2^ did not change expression of housekeeping proteins such as β-tubulin, or GAPDH. Overall our data indicated that extracellular-acting endogenous Hi-FGF-2 promotes or sustains the activated fibroblast state.

**Figure 8:**
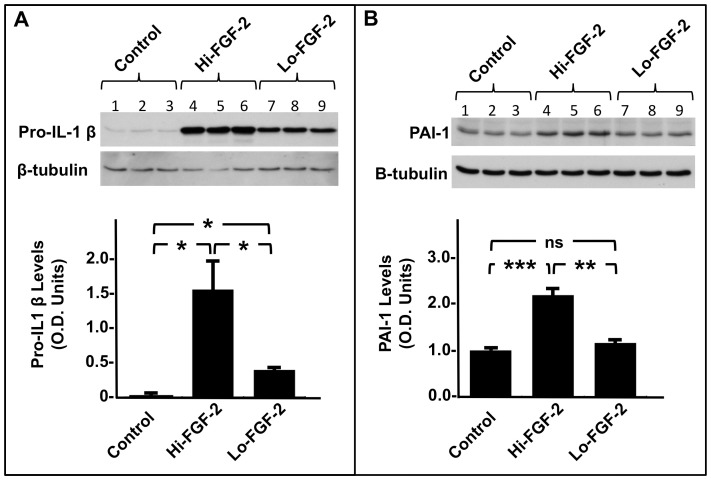
Effect of extracellular-acting FGF-2 isoforms on the accumulation of pro-IL-1β and PAI-1 by hMFs. **Panel A**, western blot and corresponding cumulative data showing relative pro-IL-1β levels (optical density, O.D. units) in hMF cell lysates from unstimulated cells (lanes 1,2,3) and cells stimulated with 10 ng/ml of a recombinant Hi-FGF-2 preparation (Hi, lanes 4,5,6) or 10 ng/ml of recombinant Lo-FGF-2 (Lo, lanes 7,8,9), as indicated. Both Hi- and Lo-FGF-2 upregulated pro-IL-1β, although the effect of Hi-FGF-2 was significantly more potent. **Panel B**, western blot and corresponding quantitative data showing relative PAI-1 levels (optical density, O.D. units) in hMF cell lysates from unstimulated cells (lanes 1,2,3) and cells stimulated with Hi-FGF-2 (Hi, lanes 4,5,6) or Lo-FGF-2 (Lo, lanes 7,8,9), as indicated. Hi- but not Lo-FGF-2 upregulated PAI-1 levels. Brackets mark comparisons between groups where *, **, ***, and ns denotes P<0.05, P<0.01, P<0.001, and P>0.05 respectively.

Cells (hMFs) were treated with preparations of recombinant human Hi- or Lo- FGF-2 at 10 ng/ml each and examined for accumulation of pro-interleukin-1β (pro-IL-1β) as well as plasminogen activator inhibitor 1 (PAI-1), proteins linked to inflammation. As seen in Fig.8A, Hi-FGF-2 elicited a robust upregulation of pro-interleukin-1β (pro-IL-1β), compared to unstimulated cells. Lo-FGF-2 also stimulated pro-IL-1β expression, but was significantly less potent (by 5-fold) compared to Hi-FGF-2; Fig. 8A. Hi-FGF-2 also promoted PAI-1 upregulation while Lo-FGF-2 had no effect; Fig. 8B.

**Figure 9:**
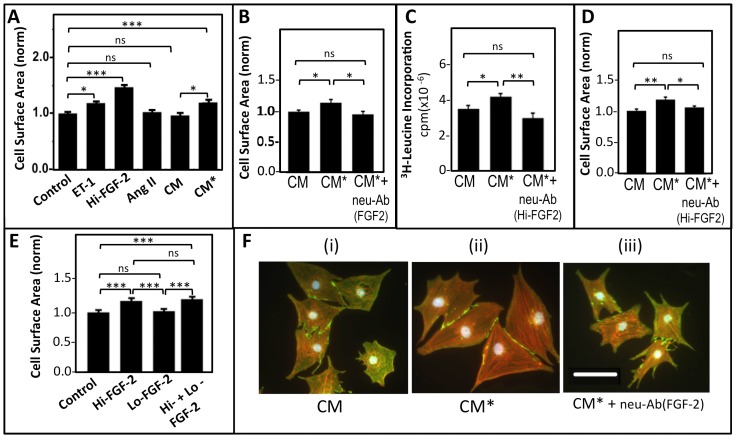
Human Hi-FGF-2 exerts pro-hypertrophic effect. **Panel A.** Neonatal rat cardiomyocyte cell surface area (normalized, assigning a value of 1 in control, untreated samples) is shown in response to stimulation with Endothelin 1 (ET-1), serving as a positive control, and a recombinant human Hi-FGF-2 preparation (10 ng protein/ml), n = 320 myocytes/group. CM denotes conditioned medium obtained from unstimulated hMFs while CM* denotes conditioned medium from Ang II-stimulated hMFs. ET-1, recombinant human Hi-FGF-2, as well as CM* (but not CM, or Ang II added at 100 nM) increased myocyte cell surface area significantly. **Panel B**. Cardiomyocyte cell surface area (normalized) is shown as a function of incubation with CM, CM*or CM* supplemented with neutralizing antibodies to total FGF-2 (neu-Ab^FGF-2^), as indicated; n = 480 cells/group. Neutralization of total FGF-2 eliminated the ability of CM* to increase myocytes cell surface area compared to CM. **Panel C.** Protein synthesis (^3^H-Leucine incorporation) of cardiomyocytes incubated with CM, CM*, and CM* supplemented with 20 µg/ml neutralizing anti-Hi-FGF-2 antibodies (CM* +neu-Ab^Hi-FGF-2^). Neutralization of Hi-FGF-2 eliminated the ability of CM* to increase protein synthesis of cardiomyocytes compared to CM; n = 5 plates/group. **D**. Cardiomyocyte cell surface area (normalized) is shown as a function of incubation with CM, CM*, and CM* +neu-Ab ^Hi-FGF-2^. Neutralization of Hi-FGF-2 eliminated the ability of CM* to increase surface area of cardiomyocytes compared to CM; n = 480/group. Please note that for the experiments shown in B,C,D panels the conditioned media in the first two groups (CM, CM*) were supplemented with non-specific rabbit IgG, at 20 µg/ml. **E**. Representative images of cardiomyocytes stained for anti-N-cadherin (green), alpha-actinin (red) and nuclei (blue), and incubated with CM, CM*, and CM* +neu-Ab (FGF-2). Sizing bar in (iii) coresponds to 100 µM. In all graphs, brackets show comparison between groups, where *, **, ***, ns correspond to P<0.05, <0.01, <0.001, or P>0.05.

Rodent Hi-FGF-2, rather than Lo-FGF-2, has been shown to promote cardiomyocyte hypertrophy[17]. To test if human Hi-FGF-2 (recombinant, or secreted in conditioned medium) is pro-hypertrophic, we used rat neonatal cardiomyocytes, a widely used *in vitro* model of hypertrophy. Stimulation of these cells with the known pro-hypertrophic agent endothelin-1 (ET-1) was used as a positive control, and elicited a significant increase in cell size (Fig. 9A). A preparation of recombinant human Hi-FGF-2 (10 ng/ml) also promoted a significant increase in cardiomyocyte cell surface area, indicating that the human protein is indeed capable of pro-hypertrophic activity. To test for the effect of secreted human Hi-FGF-2 on hypertrophy we used conditioned media from Ang II-stimulated hMFs (CM*), in comparison to those from unstimulated hMFs (CM). As has been shown in Fig. 4, the Hi-FGF-2 content of CM* is significantly higher than that of CM. Fig. 9A shows that CM*, but not CM, significantly increased cardiomyocyte cell surface area. This effect was not due to residual Ang II activity, since direct stimulation with Ang II had no significant effects on cardiomyocyte cell size, Fig.9A. In a separate experiment (Fig.9B), the stimulatory effect of CM* on cell size was confirmed to be FGF-2-dependent: incubation with Neu-Ab^FGF-2^ (interacting with all FGF-2 isoforms) significantly decreased the effect of CM* to levels similar to those of CM. Incubation with antibodies selective for Hi-FGF-2 prevented the pro-hypertrophic effect of CM*: Neu-Ab^Hi-FGF-2^ reduced the ability of CM* to promote ^3^H-leucine incorporation (protein synthesis), Fig. 9C, or to increase cell surface area, Fig. 9D, to levels not significantly different to CM. The neutralizing effect of Neu-Ab^Hi-FGF-2^ is expected to result from the ability of this antibody to bind and sequester native human Hi-FGF-2 in CM* in a manner similar to Neu-Ab^FGF-2^. As we have shown in Fig. S4C, Neu-Ab^Hi-FGF-2^ does not interact with Lo-FGF-2.

Because Hi- and Lo-FGF-2 isoforms were found to co-exist in cell and tissue extracts, we asked if Hi-FGF-2 would be able to exert a pro-hypertrophic effect in the presence of at least equivalent levels of Lo-FGF-2. Cardiomyocytes were stimulated with preparations of human recombinant Hi-FGF-2, or Lo-FGF-2 preparations, at 10 ng/ml each, or with both Hi- and Lo-FGF-2. Lo-FGF-2 did not increase cell size, confirming our previous reports [17], [19], Fig.9E. Cardiomyocytes stimulated with either Hi-FGF-2 alone, or with both Hi- and Lo- FGF-2 showed a significant and similar increase in cell surface area compared to unstimulated cells or cells stimulated with Lo-FGF-2 only; Fig.9E. Representative images of neonatal cardiomyocytes treated with CM, or CM* (±Neu-Ab^FGF-2^) are included in Fig. 9F.

It should be noted that our preparations of human recombinant Hi-FGF-2 were found to also contain 12-16 kDa fragments from the N-terminal ‘half’ as well as a 14.5 kDa fragment from the C-terminal ‘half’ of the molecule (Fig.S4A,D); thus the actual concentration of intact Hi-FGF-2 is lower than the10 ng/ml determined for the whole preparation. The N-terminal containing fragments are not expected to exert biological effects as they do not contain the FGF-2 receptor binding site which is located towards the C-terminal half of the molecule [43]. The 14.5 kDa C-terminal fragment on the other hand may have Lo-FGF-2-like activity, as it contains most of the core Lo-FGF-2 sequence; even if that were the case we do not think that it would interfere with the activity of the intact human Hi-FGF-2, because, as shown in Fig.9E, the Hi-FGF-2 preparation retained pro-hypertrophic activity in the presence of added Lo-FGF-2.

## Discussion

The high molecular weight isoforms of FGF-2 have been largely ignored in studies regarding human cardiac pathology. This is likely due to the presumption that Hi-FGF-2 (unlike Lo-FGF-2) is not amongst the cytokines/growth factors that are secreted to the extracellular space and mediate tissue remodelling by an autocrine and/or paracrine mechanism. The present study aims to challenge this perception by drawing attention to the expression and potential pathological role of Hi-FGF-2 in the human heart. We have shown, for the first time, that human Hi-FGF-2 (i) represents a substantial fraction of total FGF-2 in either atrial tissue, or in pericardial fluid, (ii) is exported to the extracellular space by hMFs, (iii) is upregulated by Ang II via both AT-1R and AT-2R receptor-activated pathways, and (iv) can exert autocrine (pro-fibrotic, pro-inflammatory) and paracrine (pro-hypertrophic) biological activities. Human Hi-FGF-2 emerges therefore as a likely contributor to maladaptive cardiac remodelling *in vivo*.

### Hi-FGF-2 in the human atria

The presence of Hi-FGF-2 in human atrial tissue extracts was documented by western blotting-based detection of 22-22.5 and 24 kDa immunoreactive bands, in addition to the 18 kDa Lo-FGF-2, using well-characterized antibodies capable of detecting all FGF-2 isoforms. The sizes of immunoreactive bands were identical to those described in the literature[35], [44]. All patient tissue extracts examined (n = 60) contained both Hi- and Lo-FGF-2 isoforms, although the relative contribution, as well as absolute amount, of each type of isoform varied considerably between individuals. At this stage, and taking into account the relatively small number of samples analyzed, no attempt was made to investigate a potential relationship between isoform distribution/concentration and patient age, gender, history, pathology or medications; a larger scale, targeted study will be required to address these issues.

Immunolocalization using anti-human Hi-FGF-2-specific antibodies corroborated the western blotting data, and in addition showed that this protein was present in atrial cardiomyocytes, interstitial, fibroblastic cells, endothelial cells, and vimentin-positive cells at or near the epicardium. All of these cells are expected to contribute to the Hi-FGF-2 content of tissue lysates. Detection of Hi-FGF-2 in the cytosol of cardiomyocytes *in vivo*, and as discussed later, in the cytosol of cardiac myofibroblasts *in vitro*, goes against the commonly held notion that Hi-FGF-2 is an exclusively nuclear protein[12], [45], but is consistent with a potential to be exported by the expressing cells. Cardiac muscle cells are known to release FGF-2 to the extracellular space on a beat-to-beat basis, through transient disruptions of their plasma membrane [46]. Detection of Hi-FGF-2 in the cytoplasm of atrial cardiomyocytes suggests that these cells might be a source of exported Hi-FGF-2 *in vivo*.

Presence of Hi-FGF-2, in addition to Lo-FGF-2, in human atria raised the question of functional roles of the different isoforms. Lo-FGF-2 is well documented to exert mitotic, cytoprotective, as well as angiogenic effects in animal models as well as humans [47]. To begin addressing the function, as well as regulation, of human Hi-FGF-2, a series of *in vitro* experiments were conducted investigating the expression and secretion of Hi-FGF-2 (compared to Lo-FGF-2) by cells such as hMFs that play a central role in tissue remodelling. Potential biological activities of extracellular-acting Hi-FGF-2 relating to tissue remodelling were also investigated.

### Expression of human Hi-FGF-2 in hMFs

Fibroblasts are responsible for the production and homeostasis of the extracellular matrix in normal tissue. Various stress stimuli, such as chronic adrenergic or neurohumoral stimulation, as well as ischemia and reperfusion damage, promote the transformation of fibroblasts to the hyper-synthetic, hyper-secretory and hyper-contractile myofibroblast phenotype. While myofibroblasts are important in cardiac repair and scar formation, persistent presence of these cells plays a central role in maladaptive remodelling and eventual failure [1], [2]. The primary hMF cultures used in the present study simulate the ‘activated fibroblast’, myofibroblast, phenotype [34].

Human myofibroblasts were found to express predominantly Hi-FGF-2, at over 80% of total FGF-2. All human Hi-FGF-2 isoforms, namely the 22, 22.5, 24 and 34 kDa proteins were produced by hMFs. The 34 kDa FGF-2 is a uniquely human isoform, which has not been detected previously in primary, non-transformed cells [35], [44]. It is possible that expression of 34 kDa FGF-2 occurs preferentially in human myofibroblasts, as it was not detected in primary human endothelial cells. The functional role of 34 kDa FGF-2 is currently unknown, although overexpression studies have suggested that it enhances cell survival[35] [44].

### The role of Ang II signaling in human Hi-FGF-2 accumulation by hMFs

Ang II upregulated human Hi-FGF-2 accumulation in hMFs, suggesting that Hi-FGF-2 may contribute to the diverse cardiac pathologies associated with Ang II elevation *in vivo*. Chronic activation of the renin angiotensin system causes maladaptive cardiac remodelling including fibrosis and hypertrophy in ventricles as well as atria[48], and is managed by angiotensin converting enzyme (ACE) inhibitors and/or Ang II receptor AT-1 antagonists[49]. The beneficial effects of ACE inhibitors and AT-1R receptor antagonists on patients could be attributed, to some extent, to a reduction in Hi-FGF-2, as losartan attenuated the Ang II-induced human Hi-FGF-2 upregulation. Nevertheless, as shown here, AT-2R was also implicated in the Ang II-induced Hi-FGF-2 upregulation, and would be expected to sustain elevated Hi-FGF-2 expression even when AT-1R is blocked. The role of AT-2R in cardiac pathology remains insufficiently understood; it is generally believed that, unlike AT-1R, AT-2R has beneficial effects counteracting several of the effects triggered by AT-1R [50], [51]. On the other hand, there is some evidence that AT-2R may exert similar effects as AT-1R, by mediating left ventricular hypertrophy and fibrosis in Ang II-induced hypertensive disease[52]. It is of interest that unlike AT-1R, AT-2R levels are elevated in the fibrillating and fibrotic atria and in the failing heart[53], [54], a situation that might perpetuate a pathology-inducing stimulus by upregulating Hi-FGF-2.

The signal transduction pathway(s) leading to increased human Hi-FGF-2 accumulation downstream of Ang II/AT-1R or Ang II/AT-2R remain to be determined. While both AT-1R and AT-2R belong to the G protein-coupled receptor superfamily, they are known to activate different downstream pathways. AT-1R is linked to growth factor signaling pathways, requiring tyrosine kinase receptors, and kinase-driven phosphorylations, while AT-2R is linked to activation of several types of phosphatases that are believed to counteract the phosphorylation events induced by AT-1R; for a detailed description of Ang II receptor signaling pathways the readers are referred to [24]. The present study shows that the Ang II-induced Hi-FGF-2 upregulation requires the ERK activating pathway, in agreement with previous work regarding expression of FGF-2 of unknown isoform composition by cardiac fibroblasts [7], [55]. In addition, both AT-1R and AT-2R were found to mediate the Ang II-induced ERK activation. While AT-1R has been linked to the Ang II-induced activation of ERK [56], AT-2R is reported to inhibit the AT-1R-induced ERK activation [57]. In other cell models, AT-2R is reported to activate the ERK pathway[58], in agreement with our present findings. Endogenous FGF-2 expression would also be expected to contribute to overall levels of activated ERK in hMFs via both intracrine and autocrine routes, as we have documented in previous studies[25]. In agreement, inhibition of extracellular-acting FGF-2 in our system decreased the magnitude of Ang-II-associated ERK activity, which decreased further, and significantly, with concurrent inhibition of AT-1R and/or AT-2R. We suggest that the ability of Ang II to upregulate FGF-2/Hi-FGF-2 requires ERK activation which occurs downstream of AT-1R and/or AT-2R as well as downstream from extracellular-acting FGF-2.

Inhibition of MMP activity was found to prevent the Ang II-induced Hi-FGF-2 upregulation in hMFs, but the timing and immediate targets of MMP action are not known. MMP activity is reported to mediate the Ang II-induced secretion of several cytokines (IL-6, IL-1β, tumor necrosis factor α, transforming growth factor β) by cardiac fibroblasts [59]; and FGF-2 release from the extracellular matrix of the lens [40]. It remains to be determined whether MMP inhibition prevented the release of cytokines and growth factors, including FGF-2, by hMFs, and therefore blocked an autocrine and/or auto-stimulatory component in the Ang II-induced FGF-2, and Hi-FGF-2 upregulation.

### Secretion/release of human Hi-FGF-2

We have shown for the first time that human Hi-FGF-2 is released to the extracellular environment by cardiac cells, hMFs, *in vitro*, and that Ang II elicited a significant increase in exported Hi-FGF-2, which seemed to parallel the increase in total cell-associated Hi-FGF-2. In rat myofiboblasts, export of Hi-FGF-2 was shown to require the activity of caspase-1 [19], so it is plausible that a similar mechanism operates in human cells.

Detection of Hi-FGF-2 in patient pericardial fluid (Fig.6C) demonstrated that, in addition to Lo-FGF-2, Hi-FGF-2 can also be released or secreted by cells *in vivo*. This is in apparent contrast to a previous report that only the 18 kDa Lo-FGF-2 is secreted into the pericardial fluid[30]. A likely explanation for the discrepancy may be inadequate prevention of proteolysis which can occur even when FGF-2 is bound to heparin-sepharose, and which converts Hi-FGF-2 to a Lo-FGF-2-like protein, by truncating the N-terminal extension [32]. The ELISA-based approaches widely used for measuring FGF-2 in biological fluids do not distinguish between isoforms.

The exact cellular source of Hi- and Lo-FGF-2 in pericardial fluid is not currently known. The pericardial fluid in addition to being a passive ultrafiltrate of plasma, can also reflect the composition of cardiac interstitium, and the local production of bioactive molecules (growth factors, brain natriuretic peptides) in health and disease[30], [60], [61]. In humans, the FGF-2 concentration in pericardial fluid was 20-fold higher than in serum, correlating with high FGF-2 content in atrial biopsies, suggestive of local, myocardial production[30]. Based on available information, we suggest that all cardiac cells capable of releasing Hi-FGF-2 to the interstitial space, either through injury or via a regulated export process, would be expected to contribute to the FGF-2 content and isoform composition in myocardium as well as pericardial fluid. One can extrapolate that during the development of hypertrophic and fibrotic heart disease hyper-secretory myofibroblastic cells would be likely to increase interstitial and pericardial Hi-FGF-2 content.

The function of Hi-FGF-2 in the pericardial fluid, which bathes the heart and thus allows for, potentially, a broader range of action compared to matrix-retained proteins, remains to be investigated. Based on our *in vitro* studies discussed in the following section, it is reasonable to expect that Hi-FGF-2 in pericardial fluid may contribute to cardiomyocyte hypertrophy even in the presence of Lo-FGF-2.

### Biological activity of human Hi-FGF-2

We have shown, for the first time, that human Hi-FGF-2 displays pro-fibrotic as well as pro-inflammatory potential. In primary hMF cultures it was found that endogenous, extracellular-acting human Hi-FGF-2 promotes or maintains the activated fibroblast phenotype, and therefore can be considered as pro-fibrotic. Treatment of hMFs with neu-Ab^Hi-FGF-2^, expected to blunt the autocrine action of Hi-FGF-2, but not Lo-FGF-2, elicited significant decreases in α-SMA, EDA-Fibronectin, SMemb, and procollagen, without affecting housekeeping proteins. Alpha-SMA represents the quintessential marker of myofibroblast phenotype[62], while EDA-Fibronectin in the extracellular matrix is considered pro-fibrotic and pro-inflammatory[63]. Decreased levels of α-SMA when the extracellular action of Hi-FGF-2 is neutralized suggests that endogenous Hi-FGF-2 stimulates α-SMA accumulation in an autocrine fashion. This finding is in contrast with the effect of extracellular-acting Lo-FGF-2, reported to decrease α-SMA expression in interstitial cells [64] [65] and represents a novel, distinct activity for Hi- compared to Lo-FGF-2.

Another novel finding is the ability of extracellular-acting recombinant Hi-FGF-2 to robustly upregulate proteins linked with innate inflammation such as pro-IL-1β, and PAI-1. IL-1β is an early mediator and prominent marker of inflammation [3]. IL-1β is expressed as inactive precursor, pro-IL-1β, which is then converted to the mature, active form by the inflammasome-activated cysteine protease caspase-1[66]. Kawaguchi and colleagues have demonstrated that inflammasome activation, and hence IL-1β production, occurs in cardiac fibroblasts, not in cardiomyocytes, and represents a crucial step in the initial inflammatory response after myocardial injury[67]. Lo-FGF-2 was also found to upregulate pro-IL-1β in hMFs, however the effect was much less pronounced compared to Hi-FGF-2. In addition, unlike Hi-FGF-2, Lo-FGF-2 had no discernible effect on the accumulation of PAI-1 by hMFs. PAI-1 is a member of the serine protease inhibitor gene family and the major physiological inhibitor of the serine proteases, urokinase-type plasminogen activator, and tissue-type plasminogen activator. Increased levels of PAI-1 are implicated in a number of pathophysiological complications including fibrosis and inflammation [68]. It is of interest that Ang II has also been reported to upregulate PAI-1 expression [69], as well as IL-1β[70]; these effects of Ang II may be mediated or reinforced by the Ang II-induced Hi-FGF-2 increases shown here.

The mechanism by which extracellular-acting Hi- and Lo-FGF-2 exert differential effects on cardiac hMFs and myocytes need to be identified. Previous studies have indicated that Hi- and Lo- FGF-2 isoforms are similarly potent in activating the tyrosine kinase FGF-2 cell surface receptors (FGFR) and pathways downstream of FGFR[16]. This would suggest that differential effects are not mediated by differences in receptor binding or activation. It should, however, be noted, that the FGFR family has many members, not only products of different genes, but also products of differential splicing, and subjected to variable degrees of post-translational modifications[71]. There is as yet no information as to which FGFR isoform(s) are expressed by activated human cardiac myofibroblasts, or whether they may display isoform-specific preferences. It should also be noted that extracellular FGF-2-triggered signal transduction includes not only plasma membrane FGFR-mediated signals (which may be common between Hi- and Lo-FGF-2) but also direct nuclear signals exerted by internalized FGF-2, which may be isoform-specific. It is of relevance that Hi- and Lo-FGF-2 localize to distinct nuclear locations, and have been proposed to exert distinct affects on nuclear function [36].

Extracellular-acting human Hi-FGF-2, either recombinant or endogenously produced, was found to be pro-hypertrophic *in vitro*, similar to rat Hi-FGF-2[17];Fig.9. It is therefore reasonable to expect that just like rat Hi-FGF-2, human Hi-FGF-2, but not Lo-FGF-2, is likely to play a pro-hypertrophic role in the human heart *in vivo*. It is of interest that the relative total and Hi-FGF-2 levels in human heart-derived hMFs were found to be 4-fold higher than those of their rat counterparts, suggesting a potential for a more prominent role for Hi-FGF-2 in humans compared to rodent. We propose that selective neutralization of secreted Hi-FGF-2 in humans would be expected to prevent or attenuate fibrosis and hypertrophy without affecting beneficial (cytoprotective and angiogenic) effects of co-expressed Lo-FGF-2.

## Supporting Information

Figure S1 **Comparison of western blot signal for recombinant FGF-2 (12.5-200 pg/lane) with anti-FGF-2 signal in representative human atrial lysate samples.** Western blot showing anti-FGF-2 immunoreactivity from 9 different patients (lanes 1–9, 50 µg/lane) in comparison to the immunoreactivity elicited by recombinant histidine (*His*)-tagged low molecular weight FGF-2 loaded at 12.5, 25, 50, 100 and 200 pg/lane. Please note that due to the *His*-tag, FGF-2 migrates near 22 kDa. The anti-FGF-2 signals in the 9 patients shown are representative of the range in total FGF-2 signal, as well as relative isoform composition, encountered in all patients analyzed; intensity of the various anti-FGF-2 bands was within the selected recombinant FGF-2 range.
(JPG)

Figure S2 **Localization of Hi-FGF-2 in cardiomyocytes and non-myocytes in human atrial tissue.** **Panels A and B** show the area included within an inset in Fig.1F, stained, respectively, for Hi-FGF-2 (green) and desmin (red), and counterstained with DAPI for nuclei (blue). Yellow arrows point to nuclei staining positive for Hi-FGF-2. Pink arrows point to cardiomyocyte cytosolic compartment, also staining positive for Hi-FGF-2. **Panel C** shows a larger magnification image of the inset within Fig.1G, and represents atrial cells near the epicardial region (white arrows) staining positive for vimentin (red) and Hi-FGF-2 (green). **Panel D** shows immunohistochemical anti-Hi-FGF-2 staining of an atrial tissue section from a healthy individual. Black pointed arrows identify Hi-FGF-2-positive cells found in the epicardial lining; blue arrows identify fibroblastic connective tissue cells. Yellow arrows point to myocyte nuclei staining positive for Hi-FGF-2. Pink arrows point to cardiomyocyte cytosolic compartment, also staining positive for Hi-FGF-2. Sizing bars in B,C and D correspond, respectively to 50, 20 and 100 µM.
(JPG)

Figure S3 **Anti-human Hi-FGF-2 antibodies detect overexpressed human Hi-FGF-2 but not human Lo-FGF-2,** ***in situ.*** Human Hi- or Lo-FGF-2 were overexpressed in human embryonic kidney (HEK) 293 cells by transient gene transfer. HEK293 cells express low levels of endogenous FGF-2, allowing clear detection of transfected, FGF-2-overexpressing cells with appropriate antibodies. One day after gene transfer, cells were subjected to triple fluorescence staining with rabbit polyclonal anti-human Hi-FGF-2 (green), mouse monoclonal anti-FGF-2 (red), detecting both Hi- and Lo- FGF-2, and DAPI nuclear stain (blue). **A,B,C,D** panels show the same field from HEK293 cells overexpressing human Lo-FGF-2. A,B,C are stained, respectively, for Hi-FGF-2, total FGF-2 and nuclei, while D shows the merged image from A,B,C. Arrows point to cells overexpressing Lo-FGF-2, clearly identified by the monoclonal anti-FGF-2 antibodies (B, D). The overexpressing cells are not detected by anti-Hi-FGF-2 antibodies (A, D). **E,F,G,H** panels show the same field of HEK293 cells overexpressing human Hi-FGF-2 (22-24 kDa). E,F,G are stained, respectively, for Hi-FGF-2, total FGF-2, and nuclei, while H shows the merged image from E,F,G. Arrows point to overexpressing cells, clearly detected by both anti-Hi-FGF-2 antibodies (E, H), and anti-FGF-2 antibodies (F, H). Please note that panel D has been deliberately overexposed for ‘green’, to obtain an outline of the cell layer.
(JPG)

Figure S4 **Specificity of anti-human Hi-FGF-2 antibodies for denatured and native Hi-FGF-2. Panel A. Anti-human Hi-FGF-2 antibodies detect recombinant human Hi-FGF-2, but not rat Hi- or Lo-FGF-2, by western blotting.** *His*-tagged recombinant FGF-2 proteins, including human Hi-FGF-2 (24 kDa, migrating near 30 kDa due to the *His*-tag), rat Hi-FGF-2 and rat Lo-FGF-2, loaded at 10 and 50 ng/lane, were analyzed by western blotting, and probed with monoclonal antibodies recognizing all human and rat FGF-2 isoforms (monoclonal anti-FGF-2, raised against the 18 kDa bovine Lo-FGF-2) or polyclonal antibodies raised against a sequence specific for the N-terminal of human Hi-FGF-2 (anti-Human-Hi-FGF-2), as indicated. In the gel loaded with recombinant human Hi-FGF-2 (lanes 1, 2), both antibodies recognize a band near 30 kDa representing the intact *His*-tagged human Hi-FGF-2; anti-Hi-FGF-2 antibodies also detect fragments at 15.5 and 12 kDa, containing the N-terminal of the molecule, while anti-FGF-2 antibodies recognize a 14.5 kDa fragment, containing the C-terminal of the molecule. The anti-FGF-2 antibodies detect, as expected, rat Lo-FGF-2 (lanes 3, 4) and rat Hi-FGF-2 (lanes 5, 6); these bands are not detected by the anti-human Hi-FGF-2 antibodies. **Panel B**. Schematic linear representation of domains within the sequence of recombinant human Hi-FGF-2. N- and C- point to the N- and C-terminii of the molecule. The core Lo-FGF-2 sequence is represented by pink color, while the N-terminal extension present only in Hi-FGF-2 is represented by green; the pale green edge indicates the histidine tog present in the recombinant molecule. A blue arrow points to the epitope(s) recognized by the polyclonal anti-Hi-FGF-2 antibodies. The monoclonal anti FGF-2 antibodies recognize epitopes within the Lo-FGF-2 core sequence. **Panel C**. **Anti-human Hi-FGF-2 antibodies interact with native endogenous human 22-24 kDa Hi- (but not Lo-) FGF-2 in solution**. This western blot shows that anti-human Hi-FGF-2 antibodies specifically immunoprecipitate endogenously expressed human Hi-FGF-2 from embryonic human myofibroblasts. These cells express both Hi- and Lo-FGF-2 isoforms. The blot was probed for total FGF-2 with the monoclonal anti-FGF-2 antibodies. **Lane 1**, total lysate (20 µg) from human embryonic cardiac fibroblasts, before being subjected to immunoprecipitation (IP), contains 22-24 Hi-FGF-2 (blue arrows), 34 kda Hi-FGF-2 (asterisk) and 18 kDa Lo-FGF-2 (orange arrow). **Lane 2**, cell lysate proteins retained non-specifically by protein A-sepharose beads (IP-control) from 900 µg lysate show no immunoreactive signal. **Lane 3**, cell lysate proteins retained by the anti-Hi-FGF-2/protein-A-sepharose column (IP-anti-Hi-FGF-2) from 900 µg lysate show strong immunoreactive signal at 22-24 kDa, representing human Hi FGF-2 isoforms. No signal for the 18 kDa FGF-2, or the 34 kDa FGF-2 is present. **Lane 4**, cell lysate (20 µg) after being subjected to immunoprecipitation with anti-Hi-FGF-2 and protein-A-sepharose. Blue arrows point to the signal for 22-24 kDa Hi-FGF-2 which is reduced compared to that before immunoprecipitation (lane 1), consistent with selective removal of the 22-24 kDa isoforms from the extracts by the affinity column. A 34 kDa immunoreactive band (presumably the 34 kDa human-Hi-FGF-2), marked with an asterisk, has not been depleted from the extract by the anti-Hi-FGF-2 affinity column., even though the 34 kDa human Hi-FGF-2 contains the immunoreactive epitope(s). Because, as shown next in panel D, the anti-Hi-FGF-2 antibodies can recognize denatured 34 kDa Hi-FGF-2, inability to immunoprecipitate the native protein indicates epitope masking under non-denaturing conditions. **Panel D**. **Anti-human Hi-FGF-2 antibodies detect 34 kDa Hi-FGF-2 in its denatured state**. The heparin-sepharose-bound fraction from 1 mg of human embryonic fibroblast lysate was analyzed for Hi-FGF-2 by western blotting (**lane 5**). Recombinant human *His*-tagged 24 kDa Hi-FGF-2 (**lanes 6,7**) was used as positive control for anti-Hi-FGF-2 immunoreactivity. The anti-Hi-FGF-2 antibodies detect a 34 kDa band, indicated by an asterisk in lane 5. As expected (see panel A), in addition to detecting intact 22-34 kDa FGF-2, the anti-Hi-FGF-2 antibodies detect N-terminal containing Hi-FGF-2 fragments present in cell extracts and retained by heparin-sepharose (lane 5), or present in recombinant Hi-FGF-2 preparations (lanes 6,7).
(JPG)

Figure S5 **Identification of human patient atria-derived cells as myofibroblasts (hMFs).** **A,B,and C**. Triple fluorescence staining of cells for alpha smooth muscle actin (α-SMA, red, A, C), vimentin (green, B, C), and nuclei (blue, C). Stress fibers (α-SMA positive) are a characteristic of myofibroblasts. **D**. Close-up image of a cell subjected to triple fluorescence staining for α-SMA (red), vimentin (green), and nuclei (blue) clearly show presence of both vimentin- and α-SMA-composed filaments within the same cell, identifying it as myofibroblast. **E**. Western blot analysis of lysates from hMFs and rat neonatal cardiomyocytes (three different samples per group), probed for markers of myofibroblast phenotype (EDA-Fibronectin, SMemb, procollagen, vimentin, α-SMA) and cardiomyocyte phenotype (desmin, striated muscle myosin, Troponin-T, TnT), as indicated. Cells defined as hMFs express EDA-Fibronectin, SMemb, procollagen, vimentin, α-SMA, but not desmin, TnT, or myosin; corresponding antibodies clearly detect desmin, TnT and myosin in cardiomyocytes. Staining for Ponceau Red (P-Red) is also shown.
(JPG)

Figure S6 **Production of Hi- and Lo- FGF-2 isoforms by cardiac myofibroblasts from different sources, and endothelial cells. Panel A. Cell-Associated FGF-2.** Representative western blot images from extracts of: rat adult ventricular myofibroblasts; human adult ventricular and atrial myofibroblasts (loaded at 10 µg/lane); human embryonic ventricular myofibroblast extract (loaded at 20 µg/lane), and probed for FGF-2, as indicated. Relative migration of FGF-2 isoforms, (18, 21, 21.5 kDa for rat, and 18, 22-22.5, 24, 34 kDa for human) is shown. **Panel B.**Western blot of total lysates isolated from human lymphatic endothelial cells (n = 4, lanes 1–4), aortic endothelial cells (n = 4,lanes 5–8), and atrial-derived myofibroblasts (n = 4, lanes 9–12), at 50 µg/lane. The blot was probed with monoclonal anti-FGF-2 antibodies detecting all isoforms of FGF-2. Expression of all FGF-2 isoforms is shown to be substantially more pronounced (over 20-fold) in hMFs, compared to either type of endothelial cells.
(JPG)

Figure S7 **MMP activity is not affected by Ang II receptor activation nor extracellular-acting FGF-2. Panel A** shows gel zymograms for MMP-2 activity detected in conditioned medium from hMFs stimulated for 30 minutes with Ang II (lanes 1,2,3), Ang II + PD123319 (lanes 4,5,6), Ang II + Losartan (lanes 7,8,9), and Ang II +PD123319 +Losartan (lanes 10,11,12), in the absence (−) or presence (+) of neutralizing anti-FGF-2 antibodies (neu-Ab^FGF-2^), as indicated.The broken white line between lanes 1 and 2 indicates that these lanes were separated by more than one spaces on the gel. **Panel B** shows densitometry values (MMP Activity in arbitrary units) from the groups shown in panel A, as indicated. The vertical broken grey lines separates values obtained in the absence or presence of neu-Ab^FGF-2^ as indicated. There were no significant differences (P>0.05) between any of the groups.
(JPG)

Figure S8 **Human adult ventricular myofibroblasts export Hi-FGF-2.** Western blot-based analysis of FGF-2 content in: Lane 1, heparin-bound fraction from 60 ml of pooled conditioned medium (CM) from ventricular hMFs; lane 2, heparin-bound fraction from 60 ml medium not conditioned by ventricular hMFs (Non-CM); lane 3, 2 ng of recombinant histidine-tagged Lo-FGF-2; lane 4 is left empty, lane 5, heparin-bound fraction from a 10 ml high salt eluate containing cell-surface-associated proteins. The 22–24 kDa Hi-FGF-2 is present in CM, as well as in the cell surface-associated fraction. The 18 kDa FGF-2 is detectable only in the cell-associated fraction.
(JPG)
