# Supplementary figures and images for: High Molecular Weight Fibroblast Growth Factor-2 in the Human Heart Is a Potential Target for Prevention of Cardiac Remodeling
Source: PLoS One. 2014 May 14;9(5):e97281. doi: 10.1371/journal.pone.0097281 (PMC4020823; doi:10.1371/journal.pone.0097281)

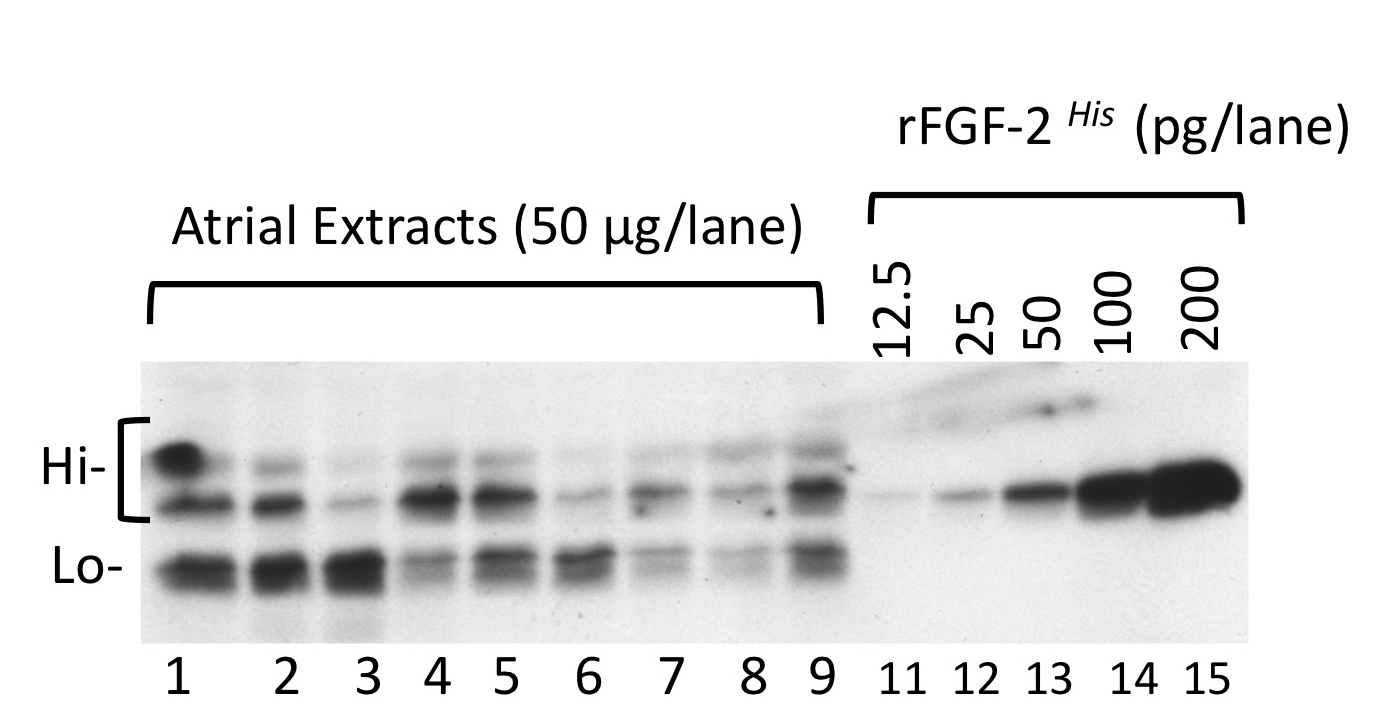

Supplement: Figure S1 — Comparison of western blot signal for recombinant FGF-2 (12.5-200 pg/lane) with anti-FGF-2 signal in representative human atrial lysate samples. Western blot showing anti-FGF-2 immunoreactivity from 9 different patients (lanes 1–9, 50 µg/lane) in comparison to the immunoreactivity elicited by recombinant histidine (His)-tagged low molecular weight FGF-2 loaded at 12.5, 25, 50, 100 and 200 pg/lane. Please note that due to the His-tag, FGF-2 migrates near 22 kDa. The anti-FGF-2 signals in the 9 patients shown are representative of the range in total FGF-2 signal, as well as relative isoform composition, encountered in all patients analyzed; intensity of the various anti-FGF-2 bands was within the selected recombinant FGF-2 range. (JPG) [file pone.0097281.s001.jpg]

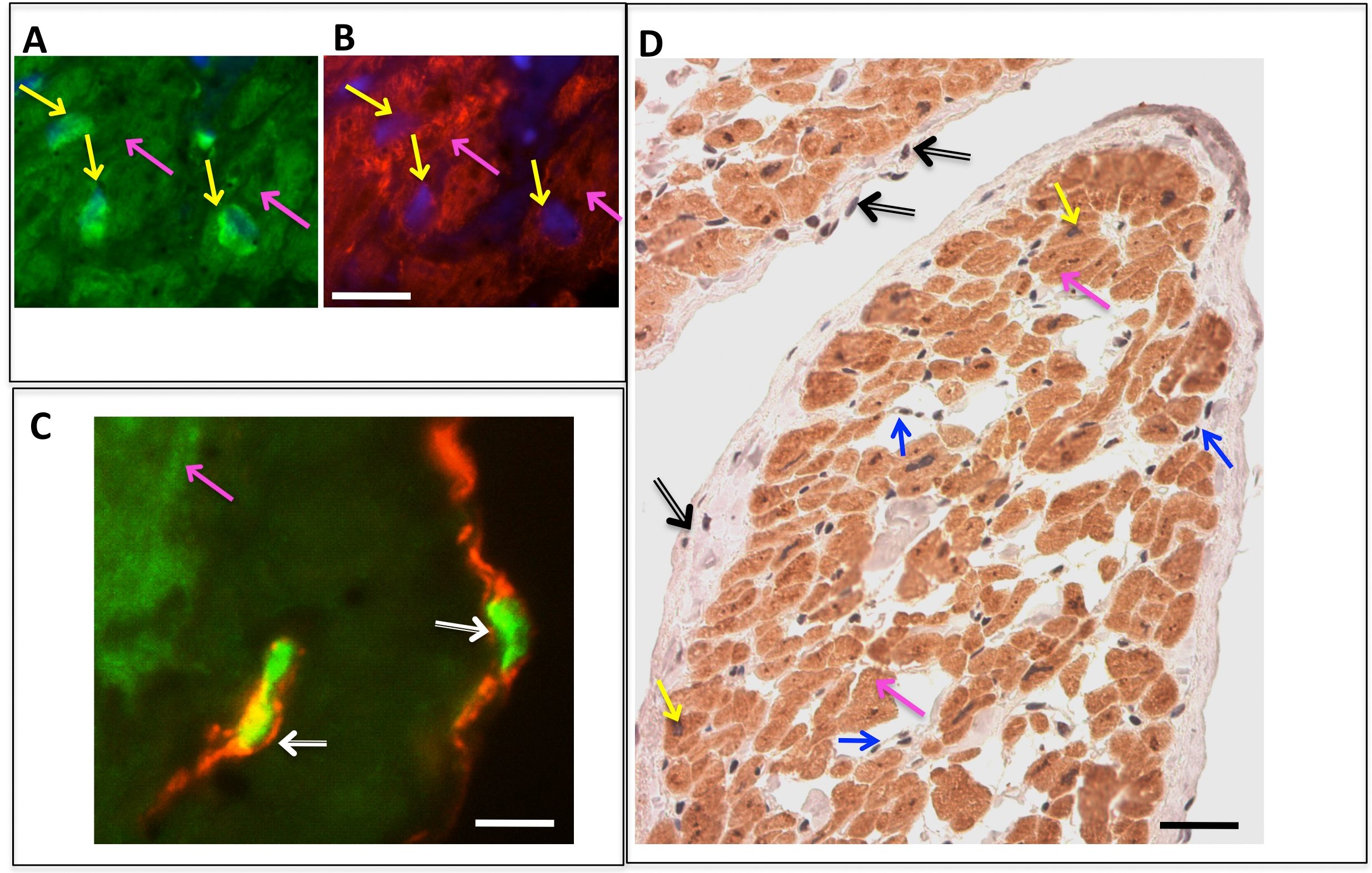

Supplement: Figure S2 — Localization of Hi-FGF-2 in cardiomyocytes and non-myocytes in human atrial tissue. Panels A and B show the area included within an inset in Fig.1F, stained, respectively, for Hi-FGF-2 (green) and desmin (red), and counterstained with DAPI for nuclei (blue). Yellow arrows point to nuclei staining positive for Hi-FGF-2. Pink arrows point to cardiomyocyte cytosolic compartment, also staining positive for Hi-FGF-2. Panel C shows a larger magnification image of the inset within Fig.1G, and represents atrial cells near the epicardial region (white arrows) staining positive for vimentin (red) and Hi-FGF-2 (green). Panel D shows immunohistochemical anti-Hi-FGF-2 staining of an atrial tissue section from a healthy individual. Black pointed arrows identify Hi-FGF-2-positive cells found in the epicardial lining; blue arrows identify fibroblastic connective tissue cells. Yellow arrows point to myocyte nuclei staining positive for Hi-FGF-2. Pink arrows point to cardiomyocyte cytosolic compartment, also staining positive for Hi-FGF-2. Sizing bars in B,C and D correspond, respectively to 50, 20 and 100 µM. (JPG) [file pone.0097281.s002.jpg]

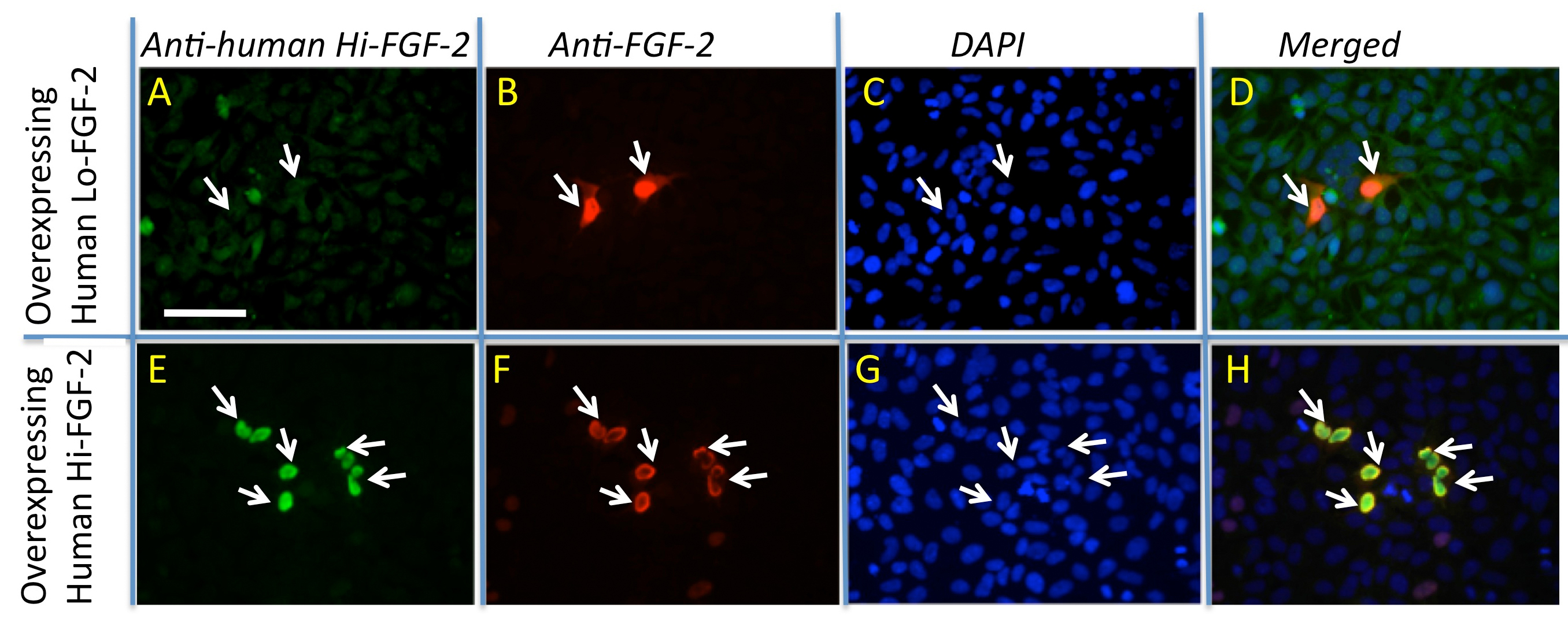

Supplement: Figure S3 — Anti-human Hi-FGF-2 antibodies detect overexpressed human Hi-FGF-2 but not human Lo-FGF-2, in situ. Human Hi- or Lo-FGF-2 were overexpressed in human embryonic kidney (HEK) 293 cells by transient gene transfer. HEK293 cells express low levels of endogenous FGF-2, allowing clear detection of transfected, FGF-2-overexpressing cells with appropriate antibodies. One day after gene transfer, cells were subjected to triple fluorescence staining with rabbit polyclonal anti-human Hi-FGF-2 (green), mouse monoclonal anti-FGF-2 (red), detecting both Hi- and Lo- FGF-2, and DAPI nuclear stain (blue). A,B,C,D panels show the same field from HEK293 cells overexpressing human Lo-FGF-2. A,B,C are stained, respectively, for Hi-FGF-2, total FGF-2 and nuclei, while D shows the merged image from A,B,C. Arrows point to cells overexpressing Lo-FGF-2, clearly identified by the monoclonal anti-FGF-2 antibodies (B, D). The overexpressing cells are not detected by anti-Hi-FGF-2 antibodies (A, D). E,F,G,H panels show the same field of HEK293 cells overexpressing human Hi-FGF-2 (22-24 kDa). E,F,G are stained, respectively, for Hi-FGF-2, total FGF-2, and nuclei, while H shows the merged image from E,F,G. Arrows point to overexpressing cells, clearly detected by both anti-Hi-FGF-2 antibodies (E, H), and anti-FGF-2 antibodies (F, H). Please note that panel D has been deliberately overexposed for ‘green’, to obtain an outline of the cell layer. (JPG) [file pone.0097281.s003.jpg]

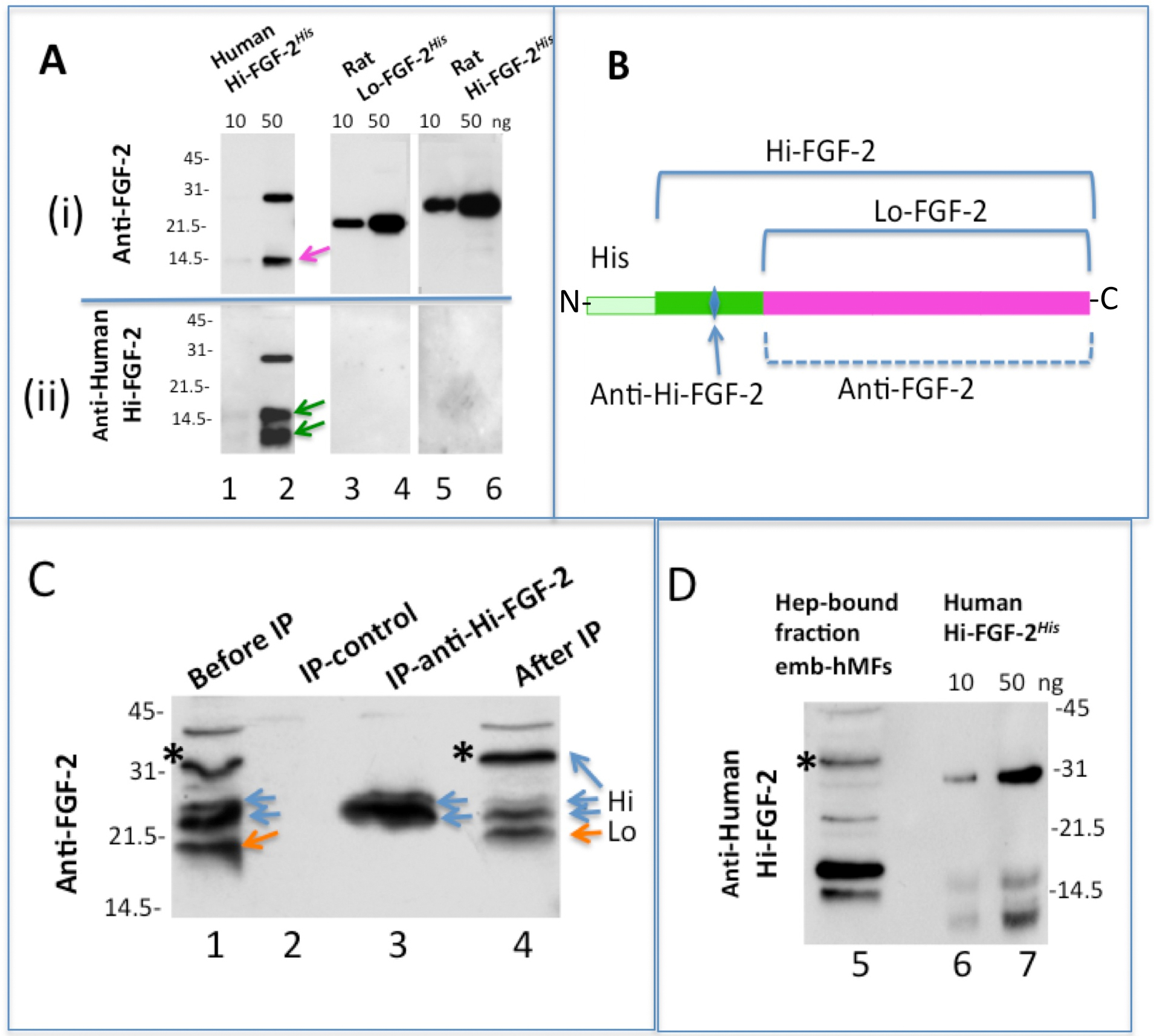

Supplement: Figure S4 — Specificity of anti-human Hi-FGF-2 antibodies for denatured and native Hi-FGF-2. Panel A. Anti-human Hi-FGF-2 antibodies detect recombinant human Hi-FGF-2, but not rat Hi- or Lo-FGF-2, by western blotting. His-tagged recombinant FGF-2 proteins, including human Hi-FGF-2 (24 kDa, migrating near 30 kDa due to the His-tag), rat Hi-FGF-2 and rat Lo-FGF-2, loaded at 10 and 50 ng/lane, were analyzed by western blotting, and probed with monoclonal antibodies recognizing all human and rat FGF-2 isoforms (monoclonal anti-FGF-2, raised against the 18 kDa bovine Lo-FGF-2) or polyclonal antibodies raised against a sequence specific for the N-terminal of human Hi-FGF-2 (anti-Human-Hi-FGF-2), as indicated. In the gel loaded with recombinant human Hi-FGF-2 (lanes 1, 2), both antibodies recognize a band near 30 kDa representing the intact His-tagged human Hi-FGF-2; anti-Hi-FGF-2 antibodies also detect fragments at 15.5 and 12 kDa, containing the N-terminal of the molecule, while anti-FGF-2 antibodies recognize a 14.5 kDa fragment, containing the C-terminal of the molecule. The anti-FGF-2 antibodies detect, as expected, rat Lo-FGF-2 (lanes 3, 4) and rat Hi-FGF-2 (lanes 5, 6); these bands are not detected by the anti-human Hi-FGF-2 antibodies. Panel B. Schematic linear representation of domains within the sequence of recombinant human Hi-FGF-2. N- and C- point to the N- and C-terminii of the molecule. The core Lo-FGF-2 sequence is represented by pink color, while the N-terminal extension present only in Hi-FGF-2 is represented by green; the pale green edge indicates the histidine tog present in the recombinant molecule. A blue arrow points to the epitope(s) recognized by the polyclonal anti-Hi-FGF-2 antibodies. The monoclonal anti FGF-2 antibodies recognize epitopes within the Lo-FGF-2 core sequence. Panel C. Anti-human Hi-FGF-2 antibodies interact with native endogenous human 22-24 kDa Hi- (but not Lo-) FGF-2 in solution. This western blot shows that anti-human Hi-FGF-2 antibodies sp [file pone.0097281.s004.jpg]

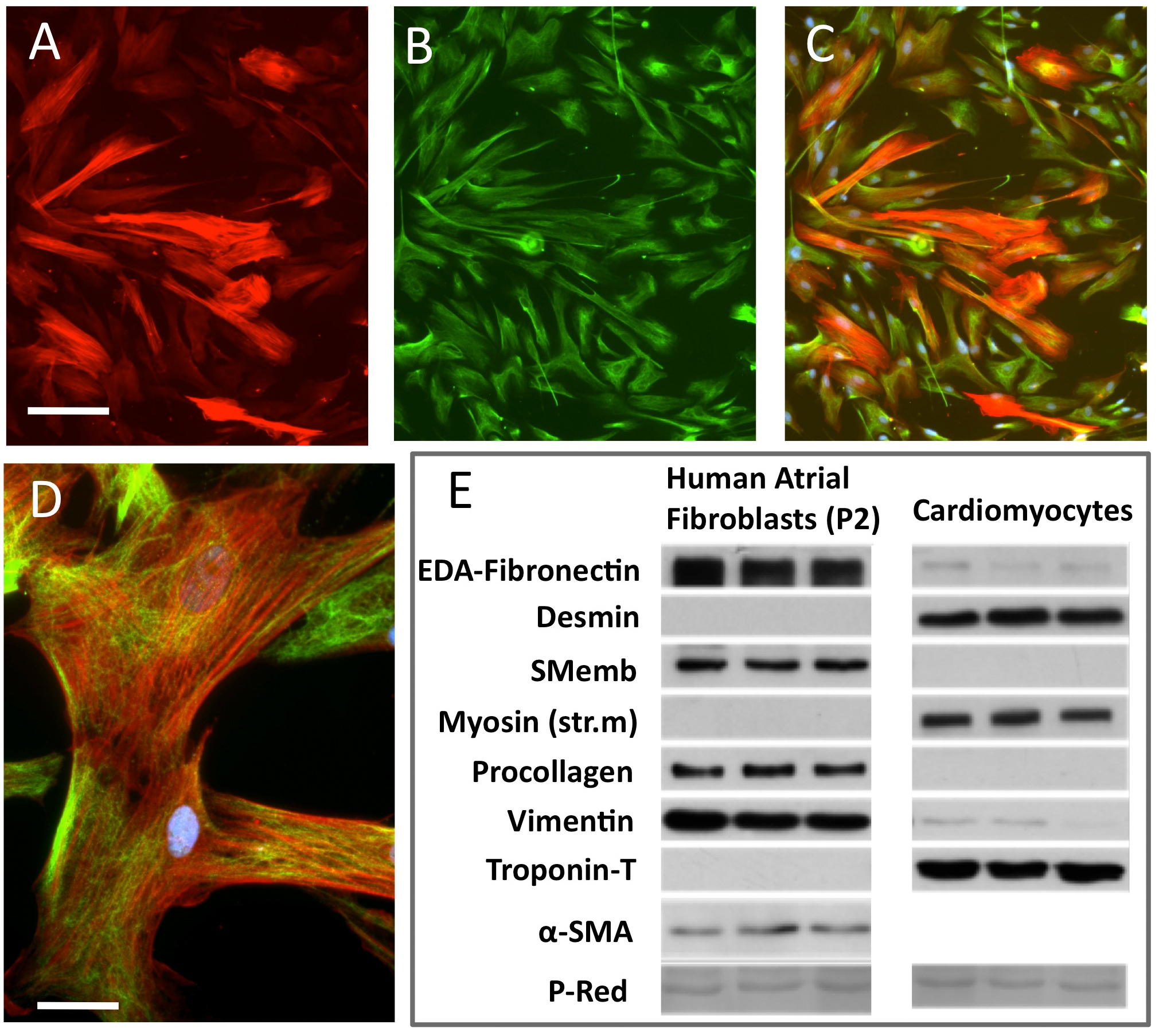

Supplement: Figure S5 — Identification of human patient atria-derived cells as myofibroblasts (hMFs). A,B,and C. Triple fluorescence staining of cells for alpha smooth muscle actin (α-SMA, red, A, C), vimentin (green, B, C), and nuclei (blue, C). Stress fibers (α-SMA positive) are a characteristic of myofibroblasts. D. Close-up image of a cell subjected to triple fluorescence staining for α-SMA (red), vimentin (green), and nuclei (blue) clearly show presence of both vimentin- and α-SMA-composed filaments within the same cell, identifying it as myofibroblast. E. Western blot analysis of lysates from hMFs and rat neonatal cardiomyocytes (three different samples per group), probed for markers of myofibroblast phenotype (EDA-Fibronectin, SMemb, procollagen, vimentin, α-SMA) and cardiomyocyte phenotype (desmin, striated muscle myosin, Troponin-T, TnT), as indicated. Cells defined as hMFs express EDA-Fibronectin, SMemb, procollagen, vimentin, α-SMA, but not desmin, TnT, or myosin; corresponding antibodies clearly detect desmin, TnT and myosin in cardiomyocytes. Staining for Ponceau Red (P-Red) is also shown. (JPG) [file pone.0097281.s005.jpg]

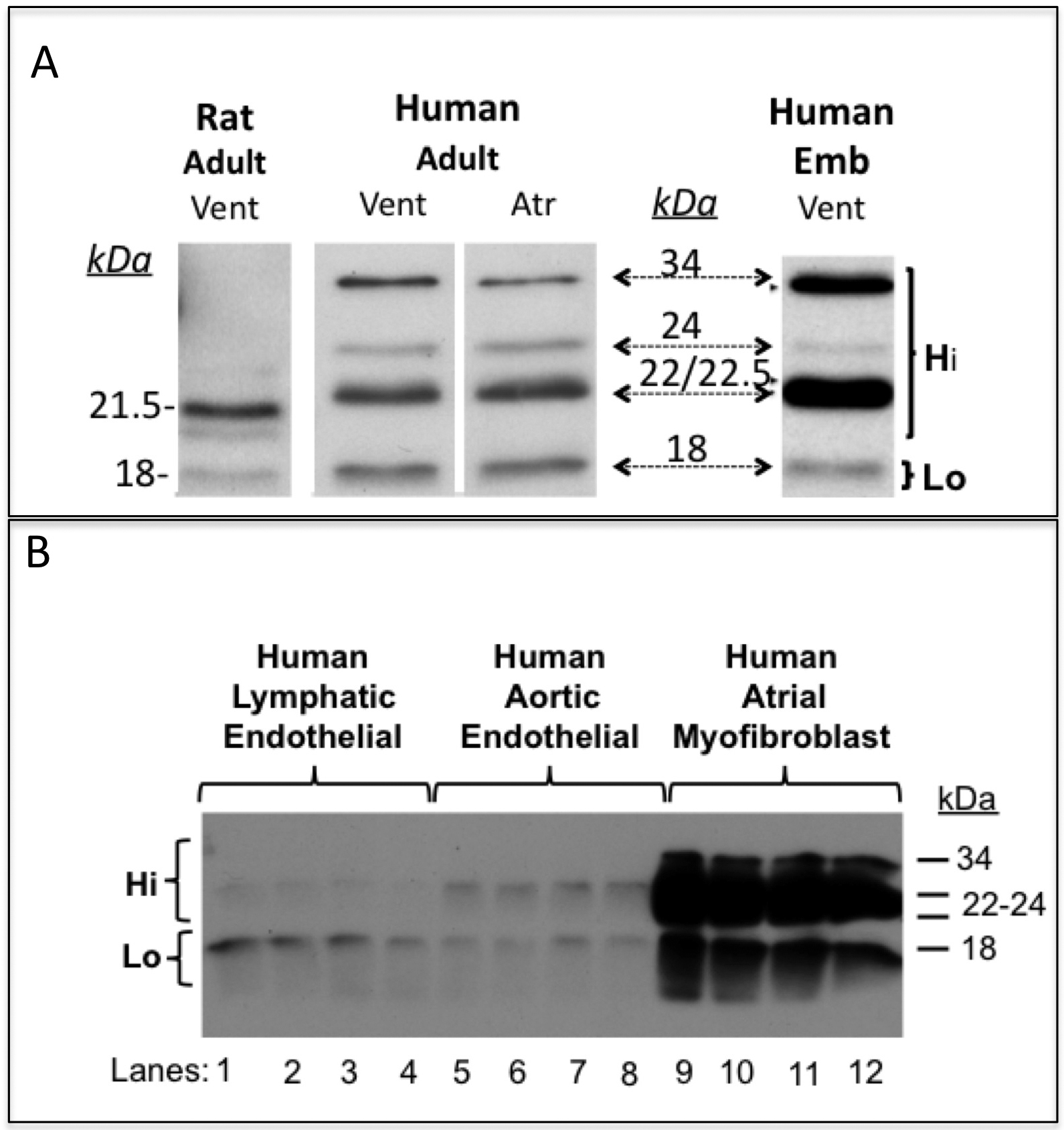

Supplement: Figure S6 — Production of Hi- and Lo- FGF-2 isoforms by cardiac myofibroblasts from different sources, and endothelial cells. Panel A. Cell-Associated FGF-2. Representative western blot images from extracts of: rat adult ventricular myofibroblasts; human adult ventricular and atrial myofibroblasts (loaded at 10 µg/lane); human embryonic ventricular myofibroblast extract (loaded at 20 µg/lane), and probed for FGF-2, as indicated. Relative migration of FGF-2 isoforms, (18, 21, 21.5 kDa for rat, and 18, 22-22.5, 24, 34 kDa for human) is shown. Panel B.Western blot of total lysates isolated from human lymphatic endothelial cells (n = 4, lanes 1–4), aortic endothelial cells (n = 4,lanes 5–8), and atrial-derived myofibroblasts (n = 4, lanes 9–12), at 50 µg/lane. The blot was probed with monoclonal anti-FGF-2 antibodies detecting all isoforms of FGF-2. Expression of all FGF-2 isoforms is shown to be substantially more pronounced (over 20-fold) in hMFs, compared to either type of endothelial cells. (JPG) [file pone.0097281.s006.jpg]

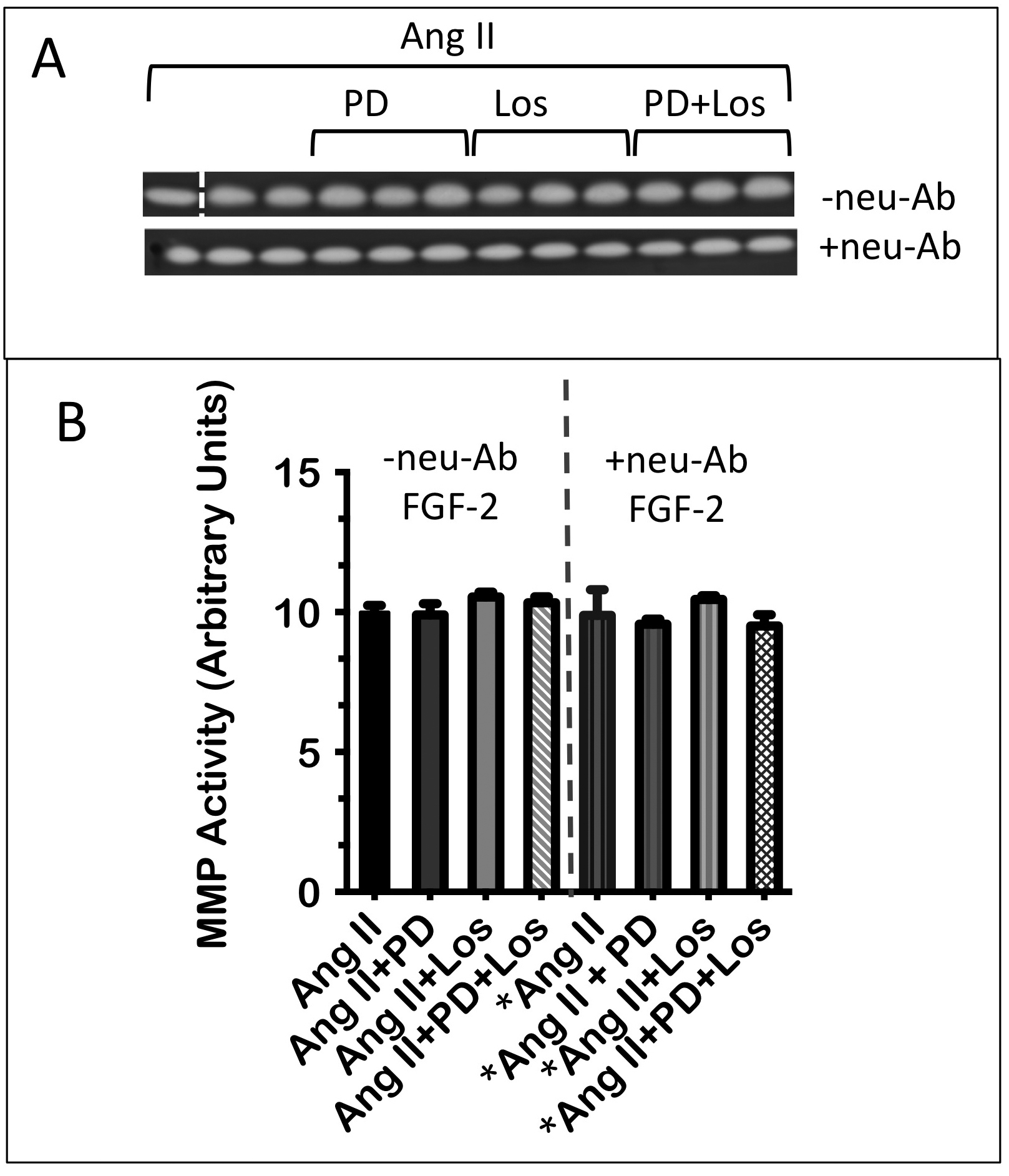

Supplement: Figure S7 — MMP activity is not affected by Ang II receptor activation nor extracellular-acting FGF-2. Panel A shows gel zymograms for MMP-2 activity detected in conditioned medium from hMFs stimulated for 30 minutes with Ang II (lanes 1,2,3), Ang II + PD123319 (lanes 4,5,6), Ang II + Losartan (lanes 7,8,9), and Ang II +PD123319 +Losartan (lanes 10,11,12), in the absence (−) or presence (+) of neutralizing anti-FGF-2 antibodies (neu-AbFGF-2), as indicated.The broken white line between lanes 1 and 2 indicates that these lanes were separated by more than one spaces on the gel. Panel B shows densitometry values (MMP Activity in arbitrary units) from the groups shown in panel A, as indicated. The vertical broken grey lines separates values obtained in the absence or presence of neu-AbFGF-2 as indicated. There were no significant differences (P>0.05) between any of the groups. (JPG) [file pone.0097281.s007.jpg]

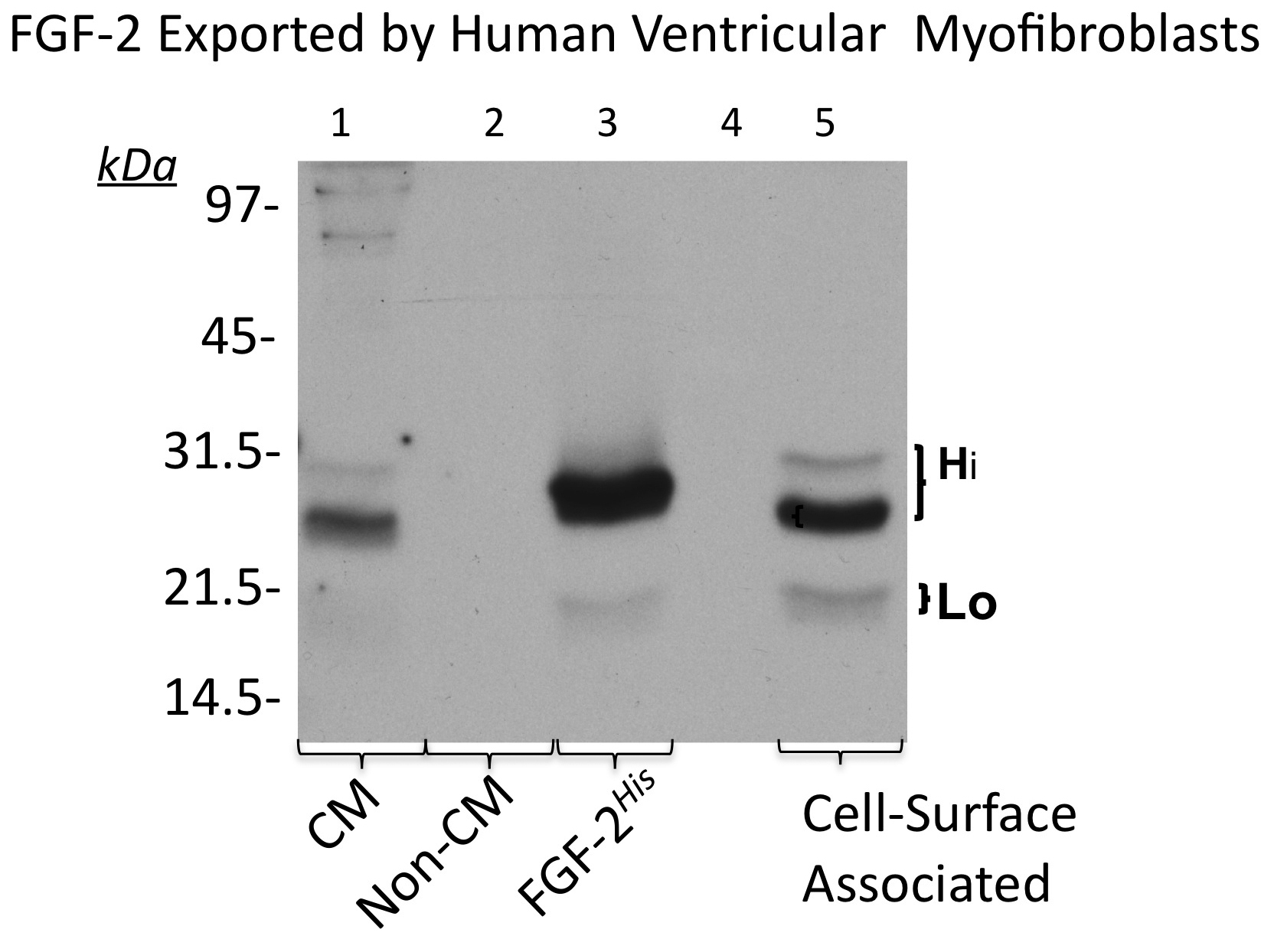

Supplement: Figure S8 — Human adult ventricular myofibroblasts export Hi-FGF-2. Western blot-based analysis of FGF-2 content in: Lane 1, heparin-bound fraction from 60 ml of pooled conditioned medium (CM) from ventricular hMFs; lane 2, heparin-bound fraction from 60 ml medium not conditioned by ventricular hMFs (Non-CM); lane 3, 2 ng of recombinant histidine-tagged Lo-FGF-2; lane 4 is left empty, lane 5, heparin-bound fraction from a 10 ml high salt eluate containing cell-surface-associated proteins. The 22–24 kDa Hi-FGF-2 is present in CM, as well as in the cell surface-associated fraction. The 18 kDa FGF-2 is detectable only in the cell-associated fraction. (JPG) [file pone.0097281.s008.jpg]
